# Identifying the motivators, benefits and barriers to sharing participant-level data and samples: results from an international online survey of acute febrile illness cohort teams

**DOI:** 10.1186/s12910-026-01399-2

**Published:** 2026-02-11

**Authors:** Priya Shreedhar, Thomas Jaenisch, Mirna Naccache, Lauren Maxwell

**Affiliations:** 1 Heidelberger Institut Für Global Health, Universitätsklinikum Heidelberg, Im Neuenheimer Feld 130/3, Heidelberg, Germany; 2 Center for Global Health, Colorado School of Public Health, University of Colorado, Denver, CO USA

**Keywords:** Acute febrile illness, Data sharing, Sample sharing, Genetic data sharing, PEARL barriers, Epidemic setting, Non-epidemic setting

## Abstract

**Background:**

Rapid and equitable sharing of de-identified, participant-level clinical-epidemiological (clin-epi) data, human biological samples, and human genetic data is crucial for an effective response to acute febrile illnesses (AFIs), particularly during epidemics. Despite increasing calls to share participant-level data and samples as part of the public health response to epidemics, several barriers continue to hinder this process. This study aimed to identify the key benefits, motivators and barriers influencing data and sample sharing among AFI cohort research teams, examining differences between epidemic and non-epidemic settings and exploring variations in perceptions across different research roles within cohort teams.

**Methods:**

We conducted a cross-sectional online survey of researchers managing AFI cohorts. Participants were recruited via cohort principal investigators (PIs) identified through global AFI research consortia and cohort lists. The survey assessed best and worst sharing experiences, perceived motivators and benefits of sharing, and barriers to sharing participant-level clin-epi data, human biological samples, and human genetic data. Best and worst sharing experiences were classified and assessed using the political, ethical, administrative, regulatory, and legal (PEARL) framework. The Wilcoxon signed-rank test was used to compare the importance of different categories of barriers between epidemic and non-epidemic settings. Analyses were also stratified by participant role (PIs vs non-PIs) to explore differences in perceptions of motivators, benefits, and barriers.

**Results:**

We received 78 responses from research teams representing 62 AFI cohorts across 23 countries. Most respondents were cohort PIs, over 45, and advanced in their careers. Most cohorts were based in South America or Central America, focused on multiple pathogens, and collected and shared multiple data types and samples. Respondents most commonly cited international scientific collaborations as the best experience and lack of benefit sharing with them as data and sample providers as the worst experience related to data and sample sharing. Important motivators and benefits included increased opportunities for collaboration, authorship, and funding, as well as enhanced insights and reduced duplication of research efforts. Regulatory and technical barriers were identified as very important in both epidemic and non-epidemic settings. Technical barriers were consistently important for both data and samples regardless of the epidemic context. Only regulatory barriers to sharing human biological samples were found to be of significantly higher importance in epidemic vs non-epidemic settings (p < 0.05). Differences emerged between PIs and non-PIs in perceived motivators, benefits; PIs more frequently highlighted authorship opportunities and funding-related collaborations as key motivators and benefits of sharing. Economic and motivational barriers to sharing were perceived to be more important by PIs than non-PIs.

**Conclusions:**

Addressing regulatory and technical barriers, particularly during epidemics, is critical for improving data and sample sharing among AFI researchers. Explicitly incorporating motivators and benefits valued by cohort researchers, including opportunities for collaboration, authorship, and funding, into data-sharing practices, alongside targeted strategies addressing differences between PIs and non-PIs, could foster more equitable and effective data and sample sharing practices.

**Supplementary Information:**

The online version contains supplementary material available at 10.1186/s12910-026-01399-2.

## Background

Sharing clinical-epidemiological (clin-epi) data, humanbiological samples, and human genetic data from health-related human subjects research is essential for informed decision-making in routine healthcare and identifying and responding effectively to public health emergencies like epidemics [1–3]. The advantages of sharing data and samples include maximising the value of the data and samples, leverage them for new insights, and translating findings more rapidly into practice, such as faster development and evaluation of diagnostics and treatments [4, 5]. Data sharing also enhances research transparency, enables verification of results, and fosters an environment of openness and collaboration within the research community [4, 6]. Data sharing reduces the burden and costs associated with unnecessary duplication of research [6]. When data are available, researchers can reuse or build upon existing studies rather than replicating them, leading to a more efficient use of resources [4, 7, 8]. Biobanks for sample storage and sharing play a critical role by providing a valuable repository of biological samples and linked clinical-epi data, which are essential for understanding complex diseases and supporting advancements in new diagnostics and treatments [9, 10].

Research funders and regulatory agencies are increasingly advocating for and require researchers to share participant-level data and biological samples equitably [11–14]. Despite the calls for increased data and sample sharing, several barriers prevent or delay sharing in epidemic and non-epidemic settings [15]. Barriers to data sharing include the absence of adequate data preservation and retrieval systems, limited use of clin-epi or genetic data standards, and resource constraints such as insufficient funding for data curation, annotation and communication [15, 1]. Researchers may also be deterred from sharing by concerns over loss of professional development opportunities (e.g., publication, grant opportunities), breaches of participant confidentiality and the associated potential to stigmatise or otherwise harm research participants. Additional barriers include experiences of lack of benefit sharing with research study teams and source populations, as well as concerns over data misuse and misinterpretation [1, 15]. Legal and regulatory barriers to data sharing include restrictive regional or national policies that limit data sharing lack of participant broad consent for future use of the data, absence of ethics review committee (ERC) or funder permission to share data, and confusion over data ownership [15, 1]. Additional barriers to sample sharing include a lack of standards for sample-related metadata, limited interoperability in sample sharing contracts, inadequate funding for preparing and transporting samples, inability to link samples to clin-epi data, insufficient mechanisms for tracking the sample chain of custody, and sharing constraints due to limited sample volume [16].

Researchers in low-and-middle-income countries (LMICs) face additional barriers to data and sample sharing stemming from the long history of colonial research, helicopter research, and other exploitative research. In these contexts, benefits of data and sample sharing for researchers and source populations, including novel disease prevention and treatments strategies and increased opportunities for publication and funding, disproportionately benefit researchers and populations in high-income countries [17–19].

To understand more about the barriers and facilitators to data and sample sharing in epidemic and non-epidemic settings, we conducted a cross-sectional survey of acute febrile illness (AFI) cohort researchers. AFIs are characterised by a sudden onset of fever, can represent myriad infectious etiologies, and are associated with high morbidity and mortality in LMICs [20, 21]. AFIs, including Zika and Ebola viruses, have caused multiple epidemics and remain a top priority for global surveillance and public health response efforts [22, 23]. Accurate and rapid diagnosis of AFIs is critical for detection and treatment but is hindered by the lack of standardised case definitions, limited access to diagnostic tools, cross-reactive or inaccurate diagnostics, and limited access to data and samples [24]. We focused the survey on AFI cohorts as improving data and sample sharing in AFI research is crucial to facilitate outbreak detection and response, accelerate the development and evaluation of diagnostics, vaccines, and treatments, identify populations at risk, better understand infection dynamics, and inform research funding priorities.

## Methods

### Study population

The target population for this study were research teams that manage cohorts that focus on pathogens associated with AFIs, including Zika virus (ZIKV), DENV, rickettsia/rickettsiosis, neorickettsia, chikungunya virus (CHIKV), Rift Valley fever virus, Mansonella perstans Filariasis, Leishmaniasis, yellow fever virus, leptospirosis/Leptospira, Japanese encephalitis virus, tick-borne encephalitis virus, hepatitis virus (any form), and Mayaro virus, as well as SARS‑CoV‑2 as cohorts expanded to address concerns surrounding the COVID-19 pandemic. Participants were recruited through lists of febrile illness cohort studies [25, 26] and from existing consortia, including the ZIKV Individual Participant Data (IPD) Consortium [27], ZIKAlliance [28], ZikaPLAN [29], AEDES Network [30], and the International Research Consortium on Dengue Risk Assessment, Management and Surveillance (IDAMS) [31]. We requested survey participation from cohort principal investigators (PIs) as well as laboratory and field data managers and other cohort staff, as the benefits and barriers to data and sample sharing may vary between these roles. We excluded cohorts focused on human immunodeficiency virus (HIV), tuberculosis (TB), malaria, or Ebola, as data and sample sharing practices within these groups may differ significantly from those in AFI cohorts focused on other pathogens. Infectious diseases (IDs) not classified as neglected tropical diseases (NTDs), such as HIV, and IDs that are well studied, like tuberculosis and malaria, often are associated with long-standing data-sharing efforts, exemplified by the WorldWide Antimalarial Resistance Network (WWARN), from which the Infectious Diseases Data Observatory (IDDO) was founded [32]. At the time of data collection, IDDO had recently developed a dedicated data-sharing platform for Ebola, potentially influencing data-sharing norms within that research community [33].

### Survey development and administration

We developed the survey through information from a systematic review of barriers to data reuse by van Panhuis et al., [15] our review of the literature on political, ethical, administrative, regulatory, and legal (PEARL) barriers to data and sample reuse, and consultation and detailed discussions with colleagues working on data and sample reuse in the context of AFIs and EIDs. We used the PEARL rather than the ethical, legal, and social issues (ELSI) approach to classifying barriers because the PEARL approach has been used previously to identify barriers to data reuse [15] and provided a more nuanced understanding of political, administrative, and regulatory barriers than the ELSI framework. To ensure clarity, comprehension, relevance, and face validity, the survey was piloted with four colleagues working with AFI cohorts, and following the pilot, the survey items were reformulated to differentiate between data and sample sharing in epidemic and non-epidemic settings to capture potential differences. We administered the one-time, online, cross-sectional survey using REDCap, a GDPR and Health Insurance Portability and Accountability Act (HIPAA)-compliant survey research platform [34]. The survey took around 20 min to complete. The full survey is available in Appendix File 2.

In August 2020, 285 AFI cohort PIs were contacted by email with the link and details of the survey. We asked PIs to forward the survey to their teams since we only had the email contact for PIs for most cohorts. We sent monthly reminders to complete the survey to the cohort PIs until December 2020. The survey covered the following areas: respondent demographic information; basic information about the AFI cohort (e.g., pathogen of interest, source population, types of data and samples collected); details of the cohort’s sharing of de-identified participant-level clin-epi data, human genetic data, and samples; open questions on the respondent’s best and worse experience sharing clin-epi data, genetic data or samples; motivators for sharing data and samples with groups outside of pre-existing partnerships with the cohorts; benefits of sharing data and samples with not-for-profit groups outside the cohort’s project or consortium; barriers to data and sample sharing both in epidemic and non-epidemic settings; details of COVID-19 data and sample sharing; information on the cohort’s inclusion of broad consent for future use of data and samples in their informed consent forms; details of the cohort’s use of data preservation, retrieval and sharing processes including the use of data standards, storage of data/samples in repositories or biobanking platforms, documented processes for external access to the cohort’s data and samples, and participation in cross-cohort data or sample sharing initiatives; and the respondent’s opinions of appropriate benefit sharing as a data provider.

### Analysis

Descriptive statistics are presented as frequencies and percentages. We conducted the statistical analyses and created the figures with R Studio version 2023.06.1 + 524.

### Barriers to data and sample sharing

In the survey, respondents used sliders to score the importance of different categories of barriers, namely “technical,” “motivational,” “economic,” “regulatory,” “legal,” “ethical,” and “political” barriers to sharing participant-level clin-epi data, human biological samples, and human genetic data in both epidemic and non-epidemic settings. These categories of barriers are based on a previously developed taxonomy of barriers to sharing data in public health by van Panhuis WG et al. [15] We considered scores between 0–33 for the barriers to be classified as “not important,” 34–67 as “somewhat important,” and 68–100 as “very important.” To assess differences in perceived barrier importance between epidemic and non-epidemic settings, we used the Wilcoxon signed-rank test, with a significance level set at α = 0.05.

### Best and worst experiences with data and sample sharing

We also asked participants to describe their best and worst experiences sharing participant-level clin-epi data, human biological samples, or human genetic data in free text fields in the survey. We then categorised responses according to the PEARL framework. Given that the PEARL framework was originally developed for categorising barriers to data sharing, we extended definitions of the “political” and “ethical” beyond those provided in van Panhuis et al. [15] for the purposes of our analysis to include categorisation of best experiences as well.

For motivators, benefits, and barriers to data and sample sharing, we compared the responses from PIs to those from non-PIs. Respondents identifying as co-investigators and investigators were grouped and classified under the PI category.

### Ethical clearance

The University of Heidelberg Faculty of Medicine's ERC approved this study’s research protocol and survey. Upon accessing the survey link, participants received detailed information about the voluntary nature of their participation, their right to withdraw, the survey's objectives, funding sources, and ethical approval. Informed consent was obtained when participants elected to begin the survey after reviewing this information.

## Results

### Characteristics of the survey respondents and cohorts

We obtained 78 survey responses, either complete or partial, from PIs and study teams representing cohorts in 23 countries. We could not determine an exact non-response rate as we asked study PIs to distribute the survey to their staff, and the total number of staff members who received the survey is unknown. Sixty-nine respondents (88%) indicated their cohort PI’s name, which we used, along with the cohort location and pathogen focus, to estimate that responses represented 62 unique cohorts. As shown in Table 1, 40 (51%) of the survey respondents were cohort PIs, 9 (12%) laboratory scientists, 6 (8%) local investigators, and 4 (5%) laboratory data managers, field data managers, and field staff or data collectors respectively, and 2 (3%) statisticians. Most respondents were 46–55 (*n* = 27; 35%) or over 55 years old (*n* = 24; 31%) and advanced in their career (*n* = 48; 62%) or mid-career (*n* = 19; 24%). More than half (*n* = 56; 72%) were from the same country where the cohort was located. About half (*n* = 45; 58%) of the cohorts were located in South America, 22 (28%) were in North or Central America, 6 (8%) each in Asia and Africa, and 2 (3%) in Europe. Over half of the respondents said their cohort (*n* = 46; 59%) focused on multiple pathogens. Twelve respondents (15%) represented cohorts focused on ZIKV only, and 7 (9%) on DENV only. Respondents indicated that the main source population for most cohorts was hospitals (*n* = 55; 71%), followed by the community (*n* = 47; 60%). Two respondents (3%) were from cohorts recruited from schools, while another two were from other sources.

**Table 1:** Survey respondent and cohort characteristics (*N* = 78)

	**N (%)**
Age range of respondents	
Under 25	1 (1)
25–35	9 (12)
36–45	15 (19)
46–55	27 (35)
Over 55	24 (31)
Not indicated	2 (3)
Respondent’s role within the cohort	
Principal Investigator/co-investigator	40 (51)
Local investigator	6 (8)
Laboratory data manager	4 (5)
Laboratory scientist	9 (12)
Field data manager	4 (5)
Field staff/data collector	4 (5)
Statistician	2 (3)
Others	7 (9)
Not indicated	2 (3)
Respondent career stage	
Early career	8 (10)
Mid-career	19 (24)
Advanced career	48 (62)
Not indicated	3 (4)
Respondent is from the country where the cohort is located	56 (72)
Cohort location	
South America	45 (58)
North or Central America	22 (28)
Asia	6 (8)
Africa	6 (8)
Europe	2 (3)
Cohort source population	
Community	47 (60)
Hospital	55 (71)
School	2 (3)
Other	2 (3)
Cohort pathogen of interest	
ZIKV only	12 (15)
DENV only	7 (9)
Multiple	46 (59)
Types of data or samples collected	
Clin-epi data only	10 (13)
Human OMICs data only	0
Human samples only	3 (4)
Multiple data or sample types	60 (77)
Collecting SARS-CoV-2 data or samples	32 (41)
Types of data or samples shared	
Clin-epi data only	22 (28)
Human OMICs data only	0
Human samples only	1 (1)
Multiple data or sample types	42 (54)
Sharing or planning to share SARS-CoV-2 data with existing platform	8 (10)
Sharing human OMICs data to a platform	7 (9)
Using a biobanking platform	20 (26)
When clin-epi data is shared	
Real-time	12 (15)
Less than or equal to 1 year after collection	18 (23)
Over one year after the collection	25 (32)
After publication	25 (32)
Other	3 (4)
When human OMICs shared	
Real-time	3 (4)
Less than or equal to 1 year after collection	2 (3)
Over one year after the collection	4 (5)
After publication	3 (4)
Other	3 (4)
When human samples are shared	
Real-time	12 (15)
Less than or equal to 1 year after collection	16 (21)
Over one year after the collection	19 (24)
After publication	13 (17)
Other	3 (4)
Do variables correspond to an internationally accepted standard	18 (23)
Consent for use of data/samples beyond the study	
Clin-epi data	29 (37)
Human samples	25 (32)
Human OMICs	7 (9)
Documented process for external groups to access data/samples	
Clin-epi data	20 (26)
Human samples	15 (19)
Human OMICs	3 (4)

Sixty respondents (77%) reported that their cohort collected multiple types of data and samples, with 32 (41%) collecting SARS-CoV-2-related data or samples. Another 10 (13%) respondents indicated that their cohort collected clin-epi data only, and 3 (4%) indicated that they only collected human-derived samples. Close to half (*n* = 42; 54%) of cohorts shared multiple data types or samples, 22 (28%) shared clin-epi data only, and one respondent indicated that their cohort shared human samples only. Eight respondents (10%) said their cohort was sharing or planning to share SARS-CoV-2 data to existing platforms, including the International Severe Acute Respiratory and Emerging Infection Consortium (ISARIC), COVID-19 Data Portal, GitHub, and COVI-PREG. Seven respondents (9%) reported that their cohort was sharing human genetic data to a platform; 20 (26%) respondents reported using a biobanking platform to make sample data visible. In terms of timelines for sharing, for clin-epi data, respondents from 12 (15%) of cohorts indicated that their cohort shared data in real-time, 18 (23%) cohorts within a one year of data collection, 25 (32%) cohorts over one year after collection, and 24 (31%) after publication. For sharing human OMICs data, respondents from 3 (4%) cohorts indicated that their cohort shared data in real-time, 2 (3%) within one year after collection, 4 (5%) over one year after collection, and 3 (4%) after publication. For the sharing of human biological samples, respondents from 12 (15%) cohorts indicated that their cohort shared samples in real-time, 16 (21%) within one year of collection, 19 (24%) over one year after collection, and 13 (17%) after publication. Twenty-nine (37%) of respondents said that their cohort obtained broad consent for future use of clin-epi data, 25 (32%) for human biological samples, and 7 (9%) for human genetic data. Twenty (26%) respondents indicated that their cohort had a documented process for external groups to access clin-epi data, 15 (19%) for human biological samples, and 3 (4%) for human genetic data. Regarding data standards, less than a quarter of survey respondents (*n* = 18; 23%) reported that the variables collected in their cohort corresponded to an internationally accepted standard. Data standards used included International Classification of Diseases (ICD)−9, 10, or 11, NCI Thesaurus, Medical Dictionary for Regulatory Activities (MedDRA), and Health Level Seven International (HL7).

### Best and worst sharing experiences

Table 2 presents respondents’ best and worst experiences sharing participant-level clin-epi data, human genetic data or human biological samples from their cohort, classified according to the PEARL framework. Fewer than one-third of survey respondents provided their best (*n* = 24; 31%) and worst sharing experiences (*n* = 18; 23%). The most commonly reported best data or sample-sharing experiences were classified as political, including eight respondents who indicated that collaborating with, exchanging ideas, and forming partnerships with experts and researchers worldwide were their best data or sample-sharing experiences. Ethical experiences, chiefly the lack of benefit sharing, including lack of communication regarding the results of the analysis of the shared data/samples to the providers (*n* = 4; 22%), not being recognised and credited in publications or otherwise (*n* = 4; 22%), and an overall lack of benefit sharing/collaboration plan (*n* = 3;17%), were the most commonly reported worst sharing experiences. Three respondents indicated that they did not have any bad experiences to share.

**Table 2:** Best and worst experiences with sharing participant-level clin-epi data, genetic data and samples^*^

	**Best sharing experiences** (*n* = 24)	**Worst sharing experiences** (*n* = 18)
Political	Collaborating with, exchanging ideas, and forming partnerships with experts and researchers across the world (*n* = 8) Contributing to a multi-national research initiative (*n* = 5) Developing high-impact scientific publications (*n* = 5) Providing data to the MoH to encourage them to formulate disease control measures (*n* = 1) Comparing results with other countries (*n* = 1)	Lack of clarity on the reason for wanting data by the research group requesting the data, especially in the case of researchers with external funding from HICs requesting data from LMICs (*n* = 1)
Ethical	Contributing to improved quality and rigour of data analysis, i.e., through larger sample size and support of a larger team of researchers (*n* = 5) Improving knowledge, awareness, and control of diseases (*n* = 3) Improving the diagnostic capabilities of different laboratories via the data sharing project (*n* = 1)	No communication regarding the results of the analysis of shared data/samples (*n* = 4) Not being recognised and credited in publications or receiving any other type of benefit for sharing data/samples (*n* = 4) Unclear or lack of benefit sharing/collaboration plan from the research group that obtained that data (*n* = 3)
Administrative	Centrally organised logistics for sample sharing (*n* = 2) Clear study plan in place (*n* = 1) Ability to contribute to analysis plans for the shared data (*n* = 1)	Lengthy duration of processes and overwhelming paperwork needed for sharing or receiving data (*n* = 2) Lack of metadata needed to prepare data for sharing (*n* = 2) Difficult to manage laboratory structures in small urban hospitals in areas with low human resources for sample sharing (*n* = 1) Not being consulted regarding data analysis plans (*n* = 1) Lack of funding to prepare the data and metadata for sharing (*n* = 1) Long duration of time needed to format data and metadata for sharing (*n* = 1)
Regulatory	Generating data to aid in the approval of diagnostics and reagents during critical periods of epidemics (*n* = 1)	Accidentally uploading images with personal identifiers to the central database (*n* = 1)
Legal	Helping to initiate conversations with sponsors to increase budgets to cover a more extensive scope of analysis (*n* = 2) Experiencing a well-defined contract in place between parties (*n* = 1)	Data recipients trying to patent a product based on the shared data/samples (*n* = 2) Suffering from lack of a well-defined contract in place between parties (*n* = 1) Long duration of time needed to obtain relevant permissions for sharing data (*n* = 1) Long duration of time required to obtain an export license for sharing samples (*n* = 1)
No good/bad experience^†^	*n* = 0	*n* = 3
No data-sharing experience was mentioned^‡^	*n* = 54	*n* = 57

^†^Explicitly stated that they did not have any good/bad experiences with sharing data or samples

^‡^Left the best and worst sharing experience fields blank in the survey, irrespective of whether or not they indicated that they shared data or samples

^*^Participants provided open text responses on their best and worst sharing experiences that were then classified according to the PEARL framework. Participants could have provided multiple experiences in their responses

### Motivators for sharing

Using pre-specified, multiple-choice options, we asked survey respondents to identify their top three motivators for sharing de-identified participant-level clin-epi data, human biological samples, and human genetic data with groups with which they did not have any pre-established relationships with (Table 3). Sharing participant-level clin-epi data for a public health rationale (other than the development of novel vaccines, treatments, or therapies) (*n* = 32; 50%), increased funding opportunities or opportunities for collaboration (*n* = 21; 33%), and conducting a cross-cohort or international study with funding support (*n* = 20; 31%) were the three most frequently chosen motivators for cohorts to share participant-level clin-epi data. Increased funding opportunities or opportunities for collaboration (*n* = 22; 41%), public health rationale (other than development of novel vaccines, treatments, or therapies) (*n* = 20; 37%), and the development of novel vaccines, treatments, or therapies (*n* = 19; 35%) were the top three motivators for cohorts to share human biological samples. Lastly, public health rationale (other than the development of novel vaccines, treatments, or therapies) (*n* = 13; 65%), the conduct of a cross-cohort or international study with funding support (*n* = 10; 50%), and the development of novel vaccines, treatments, or therapies (*n* = 5; 25%) tied with informing future research investments (*n* = 5; 25%) and increased funding opportunities or opportunities for collaboration (*n* = 5; 25%) were the top three motivators for cohorts to share human genetic data. Two survey respondents each indicated that there were no motivators for sharing participant-level clin-epi data and human biological samples. One survey respondent indicated that there were no motivators for sharing human genetic data from their cohort.

**Table 3:** Most important motivators and benefits of data or sample sharing with groups outside of pre-established partnerships with cohorts

Motivators	Benefits
De-identified, participant-level clin-epi data (***N*** = 64)	De-identified, participant-level clin-epi data (***N*** = 55)
Public health rationale, other than development of novel vaccines, treatments, or therapies (*n* = 32; 50%) Increased funding opportunities or opportunities for collaboration (*n* = 21; 33%) Cross-cohort or international study with funding support (*n* = 20; 31%) Development of novel vaccines, treatments, or therapies (*n* = 16; 25%) Cross-cohort or international study without funding support (e.g. individual participant data meta-analysis) (*n* = 14; 22%) Prevent duplication of efforts (*n* = 13; 20%) Funder requirement (*n* = 12; 19%) Inform future research investments (*n* = 10; 16%) Increased authorship opportunities (*n* = 10; 16%) National MoH requirement (*n* = 7; 11%) Local health department (state/department or regional level) requirement (*n* = 6; 9%) Moral obligation to understand the underlying pathology of diseases (*n* = 1; 2%) Obtaining insight into rare disease outcomes (*n* = 1; 2%) No motivation for sharing de-identified participant-level clin-epi data (*n* = 2; 3%**)**	Enhanced insights through collaboration (*n* = 37; 67%) Increased opportunities for authorship (*n* = 34; 62%) Increased funding opportunities (*n* = 29; 53%) Reduced duplication of efforts (*n* = 23; 42%) Long-term capacity building investment (e.g. funded postdoc) (*n* = 24; 44%) Short-term capacity building investment (e.g. short course) (*n* = 19; 35%) Free or reduced-cost access to novel diagnostics, treatments, or prevention (*n* = 14; 25%) No benefits of sharing de-identified participant-level clin-epi data (*n* = 1; 2%)
Human biological samples (***N*** = 54)	Human biological samples (***N*** = 46)
Increased funding opportunities or opportunities for collaboration (*n* = 22; 41%) Public health rationale, other than development of novel vaccines, treatments, or therapies (*n* = 20; 37%) Development of novel vaccines, treatments, or therapies (*n* = 19; 35%) Cross-cohort or international study with funding support (*n* = 18; 33%) Prevent duplication of efforts (*n* = 11; 20%) Funder requirement (*n* = 10; 19%) Increased authorship opportunities (*n* = 10; 19%) Inform future research investments (*n* = 9; 17%) Cross-cohort or international study without funding support (e.g. individual participant data meta-analysis) (*n* = 8; 15%) National MoH requirement (*n* = 6; 11%) Local health department (state/department or regional level) requirement (*n* = 3; 6%) To access better technology (*n* = 1; 2%) No motivation for sharing human biological samples (*n* = 2; 4%**)**	Expanded grant opportunities (*n* = 24; 52%) Long-term capacity building investment (e.g. funded postdoc) (*n* = 21; 46%) Free or reduced-cost access to novel diagnostics, treatments, or prevention (*n* = 20; 44%) Short-term capacity building investment (e.g. short course) (*n* = 18; 39%) Joint ownership of rights to IP produced using samples (*n* = 17; 37%) Participation in a multi-site biobanking network (*n* = 17; 37%) Infrastructure funding for biorepository (*n* = 17; 37%) Access fee (*n* = 3; 7%) No benefits of sharing human biological samples (*n* = 2; 4%)
Human genetic/OMICs data (***N*** = 20)	Human genetic/OMICs data (***N*** = 14)
Public health rationale, other than development of novel vaccines, treatments, or therapies (*n* = 13; 65%) Cross-cohort or international study with funding support (*n* = 10; 50%) Development of novel vaccines, treatments, or therapies (*n* = 5; 25%) Inform future research investments (*n* = 5; 25%) Increased funding opportunities or opportunities for collaboration (*n* = 5; 25%) Funder requirement (*n* = 4; 20%) Cross-cohort or international study without funding support (e.g. individual participant data meta-analysis) (*n* = 4; 20%) Prevent duplication of efforts (*n* = 2; 10%) Local health department (state/department or regional level) requirement (*n* = 2; 10%) National MoH requirement (*n* = 1; 5%) Increased authorship opportunities (*n* = 1; 5%) No motivation for sharing human genetic data (*n* = 1; 5%**)**	Enhanced insights through collaboration (*n* = 11; 79%) Reduced duplication of efforts (*n* = 9; 64%) Increased funding opportunities (*n* = 7; 50%) Increased opportunities for authorship (*n* = 7; 50%) Free or reduced-cost access to novel diagnostics, treatments, or prevention (*n* = 7; 50%) Long-term capacity building investment (e.g. funded postdoc) (*n* = 6; 43%) Short-term capacity building investment (e.g. short course) (*n* = 5; 36%)

**Fig. 1:**
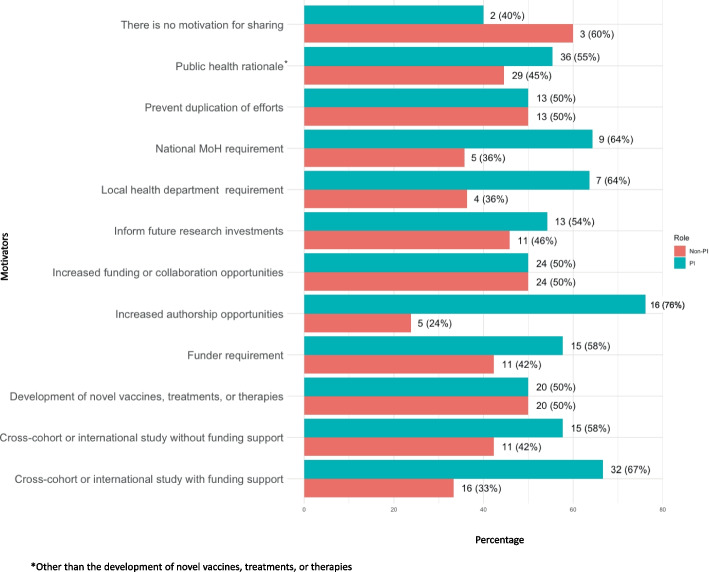
Motivators for sharing participant-level clin-epi data, human genetic data, and human biological samples for PIs and non-PIs

Figure 1 presents the motivators for sharing participant-level clin-epi data, human genetic data, and human biological samples for PIs and non-PIs. A greater percentage of PIs than non-PIs indicated that increased authorship opportunities (76% vs 24%), the ability to conduct a cross-cohort or international study with (67% vs 33%) or without funding support (58% vs 42%), funder requirement (58% vs 42%), national ministry of health (MOH) requirement (64% vs 36%), and local health department requirement (64% vs 36%) were motivators for sharing. Appendix Fig. 1A–C present the motivators for sharing participant-level clin-epi data, human biological samples, and human genetic data for PIs and non-PIs. Reflecting the overall results, compared to other study staff members, a higher percentage of PIs identified increased authorship opportunities as a motivator for sharing participant-level clin-epi data (80% vs 20%), human biological samples (70% vs 30%) and human genetic data where 100% of PIs from cohorts sharing human genetic data voted for this motivator. Funder requirements were also cited as a motivator by more PIs than other cohort staff for sharing participant-level clin-ep data (67% vs 33%) and human genetic data (75% vs 25%). The opposite was observed for sharing human biological samples, where more non-PIs than PIs saw funder requirements as a motivator (60% vs 40%). Reflecting the overall results, more PIs than non-PIs identified being able to conduct a cross-cohort study with funding support as a motivator for sharing participant-level clin-epi data (65% vs 35%), human biological samples (67% vs 33%), and human genetic data (70% vs 30%).

### Benefits of sharing

We present respondents’ top three benefits of sharing de-identified participant-level clin-epi data, human biological samples, and human genetic data in Table 3. The three most frequently chosen benefits of sharing participant-level clin-epi data were enhanced insights through collaboration (*n* = 37; 67%), increased opportunities for authorship (*n* = 34; 62%) and increased funding opportunities (*n* = 29; 53%). The three most frequently chosen benefits of sharing human biological samples from cohorts were expanded grant opportunities (*n* = 24; 52%), long-term capacity-building investment (*n* = 21; 46%), and free or reduced-cost access to novel diagnostics, treatments, or prevention (*n* = 20; 44%). For sharing human genetic data from cohorts, the three most commonly chosen benefits were enhanced insights through collaboration (*n* = 11; 79%), reduced duplication of efforts (*n* = 9; 64%), and increased funding opportunities (*n* = 7; 50%) tied with increased opportunities for authorship (*n* = 7; 50%) and free or reduced-cost access to novel diagnostics, treatments, or prevention (*n* = 7; 50%). One survey respondent indicated that there were no benefits to sharing participant-level clin-epi data, and two respondents indicated that there were no benefits to sharing human biological samples.

Appendix Fig. 2A–C present the most important benefits of sharing participant-level clin-epi data, human biological samples, and human genetic data for PIs versus non-PIs. Short-term capacity building was seen as a benefit of sharing by a greater percentage of PIs than non-PIs for clin-epi data (56% vs 44%), human biological samples (56% vs 44%), and human genetic data (60% vs 40%). Reduced duplication of efforts was also seen as a benefit of sharing by a greater percentage of PIs than non-PIs for clin-epi data (57% vs 43%) and human genetic data (56% vs 44%). For sharing human biological samples, a greater percentage of PIs than non-PIs saw joint-ownership of sample-related IP rights (65% vs 35%) and infrastructure funding for a biorepository (65% vs 35%) as benefits of sharing. Conversely, a greater percentage of non-PIs than PIs viewed participation in a multi-site biobanking network (65% vs 35%) as a benefit of sharing human biological samples. For sharing human genetic data, a greater percentage of PIs than non-PIs thought of free or reduced-cost access to novel diagnostics, treatments, or prevention (71% vs 29%) and enhanced insights through collaboration (64% vs 36%) as benefits. We were unable to combine the benefits of sharing participant-level clin-epi data, human genetic data, and human biological samples for PIs and non-PIs into one figure (as done for motivators in Fig. 1) as the benefits for sharing human biological samples that respondents could choose in the survey were different from those provided for clin-epi and human genetic data.

### Barriers to sharing

**Fig. 2:**
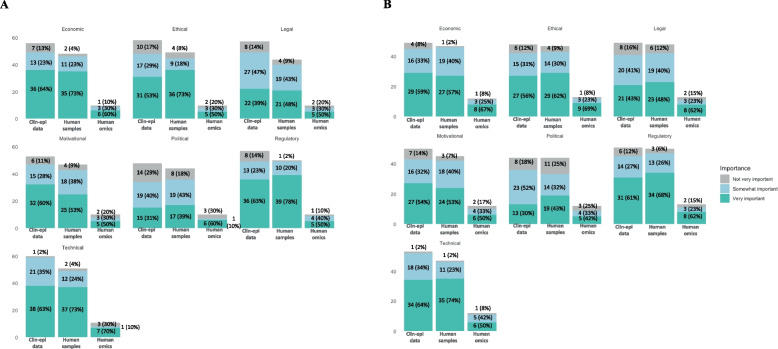
Barriers to sharing participant-level clin-epi data, human biological samples, and human genetic data within epidemic (2**A**) and non-epidemic settings (2**B**)

Figure 2A and B present respondent ratings for the different categories of barriers to sharing participant-level clin-epi data, human biological samples, and human genetic data within epidemic (2A) and non-epidemic settings (2B). Barriers most frequently scored as “very important” in epidemic settings were economic, technical and regulatory barriers to sharing participant-level clin-epi data, human genetic data and human biological samples. Technical barriers were also frequently scored as “very important” for sharing clin-epi data and human biological samples in non-epidemic settings. However, ethical barriers were more frequently scored as “very important” for sharing human genetic data in non-epidemic settings. Political barriers were most often cited as “not very important” across data and sample types in both epidemic and non-epidemic settings.

A comparison of the different categories of barriers to sharing participant-level clin-epi data, human biological samples and human genetic data between epidemic and non-epidemic settings using the Wilcoxon signed-rank test is presented in Table 4. The only category of barriers that were found to be significantly different between epidemic and non-epidemic settings were regulatory barriers to sharing human biological samples, which were scored higher in epidemic settings versus non-epidemic settings (94 vs 83; *p* < 0.05).

**Table 4:** Comparison of barriers to sharing participant-level data and samples within and outside of epidemic settings

	**Median**	***p*-value**
Participant-level clin-epi data		
Technical barriers (*n* = 52)		
Epidemic setting	75.0	0.09
Non-epidemic setting	78.0	
Motivational barriers (*n* = 45)		
Epidemic setting	75.0	0.68
Non-epidemic setting	70.0	
Economic barriers (*n*= 48)		
Epidemic setting	73.5	0.19
Non-epidemic setting	70.0	
Regulatory barriers (*n* = 48)		
Epidemic setting	87.0	0.34
Non-epidemic setting	86.0	
Legal barriers (*n* = 47)		
Epidemic setting	57.0	0.66
Non-epidemic setting	65.0	
Ethical barriers (*n* = 47)		
Epidemic setting	73.5	0.46
Non-epidemic setting	81.0	
Political barriers (*n* = 39)		
Epidemic setting	50.0	0.40
Non-epidemic setting	50.0	
Human biological samples		
Technical barriers (*n* = 46)		
Epidemic setting	81.0	0.68
Non-epidemic setting	83.0	
Motivational barriers (*n* = 42)		
Epidemic setting	70.0	0.94
Non-epidemic setting	70.0	
Economic barriers (*n* = 45)		
Epidemic setting	81.0	0.14
Non-epidemic setting	71.0	
Regulatory barriers (*n* = 46)		
Epidemic setting	94.0	0.0014*
Non-epidemic setting	82.5	
Legal barriers (*n* = 40)		
Epidemic setting	65.5	0.98
Non-epidemic setting	64.0	
Ethical barriers (*n* = 45)		
Epidemic setting	93.0	0.10
Non-epidemic setting	89.0	
Political barriers (*n* = 40)		
Epidemic setting	61.0	0.11
Non-epidemic setting	50.0	
Human genetic data		
Technical barriers (*n* = 10)		
Epidemic setting	79.0	0.94
Non-epidemic setting	69.5	
Motivational barriers (*n* = 10)		
Epidemic setting	62.5	0.29
Non-epidemic setting	67	
Economic barriers (*n* = 10)		
Epidemic setting	74.0	0.68
Non-epidemic setting	78	
Regulatory barriers (*n* = 10)		
Epidemic setting	78.0	0.83
Non-epidemic setting	94	
Legal barriers (*n* = 10)		
Epidemic setting	60.0	0.67
Non-epidemic setting	81	
Ethical barriers (*n* = 10)		
Epidemic setting	66.5	0.27
Non-epidemic setting	95	
Political barriers (*n* = 10)		
Epidemic setting	81.5	0.28
Non-epidemic setting	50	

Appendix Fig. 3 compares barriers to sharing for PIs and non-PIs and shows that a greater percentage of PIs vs non-PIs thought that economic barriers to sharing were “very important”, and that political barriers were most frequently scored as “Not very important” by both PIs and non-PIs.. Appendix Fig. 4 compares barriers for sharing data and samples separately for PIs and non-PIs and no particularly clear trends for the different categories of barriers between PIs and non-PIs were noted from this graph. Appendix Figs. 5 A and 5B compare barriers for sharing data and samples for PIs and non-PIs in both epidemic (5A) and non-epidemic settings (5B). A greater percentage of PIs than non-PIs scored economic barriers for sharing participant-level clin-epi data, human genetic data, and human biological samples as “very important” in both epidemic (clin-epi: 41% vs 23%; genetic: 40% vs 20%; samples: 42% vs 31%) and non-epidemic (clin-epi: 39% vs 20%; genetic: 42% vs 25%; samples: 36% vs 21%) settings. A higher percentage of PIs vs non-PIs scored motivational barriers in epidemic settings as “very important” for participant-level clin-epi data (34% vs 19%), human biological samples (36% vs 25%), and human genetic data (40% vs 10%).

## Discussion

We conducted a cross-sectional survey to understand the experiences, motivators, benefits and barriers to sharing participant-level clin-epi data, human biological samples, and human genetic data from AFI-related cohorts. The 78 survey responses from AFI researchers representing cohorts in 23 countries highlight diverse experiences with data and sample sharing. Most respondents were older and at an advanced career stage. Most cohorts collected multiple data types and samples; about half of the respondents reported that their cohorts had shared data or samples. Only a small proportion of respondents indicated that their cohort shared clin-epi data or human biological samples in real-time. Rapid or real-time data and sample sharing can fast-track research and development for diagnostic, treatment, and prevention-related tools in epidemic and non-epidemic settings and is especially important for addressing EIDs [2, 3, 35, 36]. Recent and recurring outbreaks, such as Ebola, Zika, and COVID-19, underscore the importance and challenges of quick, open, and effective sharing of high-quality samples and related clin-epi data and human genetic data from various populations for an informed response [37–39].

### Best and worst data and sample sharing experiences

In this study, the opportunity to form partnerships with experts and researchers worldwide was the most frequently cited best data and sample sharing experience, and is similar to findings from other studies. In a qualitative study with public health and biomedical researchers in Thailand, senior researchers highlighted the importance of sharing health research data to foster cross-national collaborations and improve their research portfolio [4]. In a survey disseminated to life scientists in 13 countries in sub-Saharan Africa, close to half of the respondents indicated that the most significant benefit of sharing their data was networking and collaboration opportunities [40].

Lack of communication regarding the results of analyses made possible through data or sample reuse and lack of benefit sharing, including not being credited in resulting publications, were the most commonly reported worst sharing experiences. The qualitative study involving Thai researchers reflects this finding, wherein researchers acknowledged that the benefits of sharing can only be realised with appropriate acknowledgement and attribution of the data providers [4]. Several researchers recounted personal experiences where data users did not adequately acknowledge them. They highlighted the harms of this, including reducing future funding opportunities, impeding career advancement, and damaging their reputation [4]. A systematic review of factors influencing data sharing also highlighted study investigator concerns around benefit sharing, which included being appropriately credited and involved in future funding opportunities made possible through data and sample reuse [41]. Another scoping review highlighted that the lack of benefit sharing that researchers from LMICs face, including being stuck as data producers only, lack of recognition, and lack of career progression, are all threats to equitable global health research [42].

### Motivators for data and sample sharing

Leading motivators for sharing participant-level data and samples included public health reasons other than the development of novel vaccines, treatments, or therapies; increased funding or collaboration opportunities; funding support for cross-study analyses; and the development of novel vaccines, treatments, or therapies. Our finding that improving public health is an important motivator for researcher’s to share data and samples is reflected in empirical research with investigators from the Ebola and Yellow Fever epidemics, wherein one participant interviewed indicated that “We share data with countries to protect them from outbreaks” [43].

We also compared the motivators for sharing participant-level clin-epi data, human biological samples, and human genetic data between study PIs and non-PIs. A substantially higher percentage of PIs found increased authorship opportunities to be a strong motivator for sharing participant-level clin-epi data, human biological samples and human genetic data compared to other study staff members. This difference may relate to different authorship opportunities within cohorts for PIs vs non-PIs [44]. PIs occupying leadership positions are likelier to see their contributions recognised and acknowledged in publications [45]. Senior researchers in Vietnam indicated that academic recognition in the form of authorship on papers that use the data they produce is crucial for them. Researchers in the same paper also saw authorship as a way of being responsible for the ethical and scientific integrity of the research [46]. These findings highlight the need to ensure that non-PIs, who often play critical roles in data generation and management, are also offered clear and equitable authorship opportunities, both within their cohorts and when data are shared externally. Doing so not only fosters a culture of fairness but also helps to overcome entrenched academic hierarchies that may otherwise discourage open collaboration and data sharing. More PIs than non-PIs for all data and sample types also considered engagement in cross-cohort or international studies with funding support as a motivator. This likely reflects the greater involvement of PIs in decisions about collaboration and external partnerships. As grant holders and study leads, PIs are more likely to be directly responsible for securing funding, managing cross-institutional relationships, and ensuring the scientific visibility of their work, making opportunities for funded international collaboration particularly attractive.

### Benefits of data and sample sharing

The top benefits for sharing participant-level clin-epi data and human genetic data indicated by survey respondents were enhanced insights through collaboration, increased opportunities for authorship, reduced duplication of efforts, and increased funding opportunities. The top benefits of sharing human biological samples included expanded grant opportunities, long-term capacity-building investments, and free or reduced-cost access to novel diagnostics, treatments, or prevention. Increased collaboration, authorship, and funding opportunities reflect the best data and sample-sharing experiences and motivators for sharing identified in this study. These have also been acknowledged to be essential benefits that need to be provided to LMIC researchers to ensure equitable data sharing in global health research [3]. Reduced duplication of research efforts has also been identified as a benefit of data sharing by other health research stakeholders, research participants, and community representatives in India and Kenya [7, 47].

We also compared the benefits of sharing participant-level clin-epi data, human biological samples, and human genetic data between study PIs and non-PIs. For all data and sample types, a greater percentage of PIs than non-PIs saw short-term capacity building as a benefit of sharing, potentially because PIs are primarily responsible for engaging with external partners and overseeing institutional development,. A qualitative study of Vietnamese researchers indicated that their view of sustainable and acceptable data sharing included reciprocity, wherein the capacity building and strengthening of data producers were prioritised [46]. These findings underscore the importance of ensuring that data sharing mechanisms are designed to deliver tangible benefits to all contributors, not only through long-term scientific impact, but also through immediate investments in skills, infrastructure, and professional development, particularly for teams in lower-resourced settings.

### Barriers to data and sample sharing

We did not find any statistically significant differences in respondent scores of different categories of barriers to sharing data or samples between epidemic vs non-epidemic settings, with the exception of regulatory barriers for sharing human biological samples which were significantly scored higher in epidemic settings versus non-epidemic settings (94 vs 83; *p*< 0.05). Similar studies on data and sample sharing in infectious disease-related observational research have identified regulatory obstacles as a primary challenge. One study examining barriers and enablers in responding to the Middle East Respiratory Syndrome Coronavirus (MERS-CoV) outbreak highlighted legal frameworks and regulatory procedures as the primary obstacles [36]. These barriers ultimately resulted in delays in MERS-CoV infection notifications, prolonged processes for data-sharing authorization, and difficulties in shipping/importing samples [36]. Similarly, another study that examined barriers to information and sample sharing in the aftermath of response efforts to combat the Zika virus outbreak highlighted regulatory delays, specifically in regulatory approvals, as one of the three significant bottlenecks faced by researchers [48].

In epidemic settings, respondents most frequently rated economic, technical, and regulatory barriers as “very important” to sharing participant-level clinical-epidemiological data, human biological samples, and human genetic data. This highlights persistent concerns about the resource demands of sharing, such as costs of data preparation, sample handling and shipment, and obtaining regulatory permissions for sharing [15, 48, 49]. The prominence of the importance of technical barriers across data and sample types as well as epidemic and non-epidemic settings reflects ongoing challenges with data standardisation and harmonisation, data preservation infrastructure limitations, as well as sample collection, handling and processing across cohorts [15, 48, 49].

Our findings also highlight differences in how various categories of barriers are perceived by cohort PIs compared to other cohort staff, across both epidemic and non-epidemic settings. Future qualitative research could explore the underlying reasons for these differences to better understand the roles, responsibilities, and incentives that shape data and sample sharing practices. Such insights could help tailor strategies to more effectively address barriers and support equitable and efficient sharing.

### Strengths and limitations

This research has several strengths. We developed the survey through consultations with AFI researchers and an extensive literature review on data and sample sharing in infectious diseases. To our knowledge, this is the first study to assess barriers and facilitators to data and sample sharing within AFI cohorts both in epidemic and non-epidemic settings and using the PEARL framework. Findings from this survey can help develop meaningful strategies for funders, cross-national surveillance systems, and other stakeholders to increase the rapidity, incidence, and quality of data and sample sharing. The survey also has several limitations. While we conducted a pilot test with colleagues working with AFI cohorts to ensure clarity, comprehension, and face validity, we did not undertake a formal psychometric validation process, such as testing internal consistency with redundant items or assessing test–retest reliability. Consequently, our survey's psychometric properties have not been formally validated, and the results should be interpreted with this limitation in mind. Most AFI cohorts were based in South, North, or Central America. However, we only circulated the survey in English, which could have prevented some respondents from answering the survey. We had more extensive lists of AFI studies and closer connections to AFI researchers from the Americas because of the recent ZIKV epidemic. AFI cohorts based in Asia and Africa were less likely to have been contacted and responded to the survey. Therefore, findings in this study cannot be assumed to be representative of all AFI research contexts nor generalisable across all countries. Results should thus be interpreted cautiously as hypothesis-generating rather than confirmatory, particularly as we could not calculate a response rate for the survey. Furthermore, as the names of the respondents and the cohorts were not collected, we had to estimate the number of cohorts that the survey respondents represented. Results should also be interpreted with caution as they could be subject to self-reported survey-related recall and response bias.

Another limitation of this study relates to our use of the PEARL framework, which has been used previously to classify barriers to data sharing in public health rather than facilitators or positive experiences with data sharing. As a result, categorising positive experiences such as international collaborations and partnerships, improved quality and rigor of data analysis, and improved diagnostic capabilities of laboratories within the existing PEARL categories required adaptation beyond the definitions provided by van Panhuis et al.

## Conclusions

Utilising a cross-sectional survey, this study identified critical motivators, benefits, and barriers experienced by AFI cohort researchers in sharing participant-level clin-epi data, human biological samples, and human genetic data. Researchers valued opportunities for collaborations or funding as important motivators and benefits of sharing; however, prior experiences of lack of benefit sharing, including insufficient acknowledgment, as well as regulatory and technical barriers to sharing in epidemic and non-epidemic settings, posed challenges. Regulatory barriers were scored significantly higher for sharing human biological samples in epidemic versus non-epidemic settings, highlighting an urgent need for streamlined regulatory processes for sharing biological samples during public health emergencies. Technical barriers remained consistently important across epidemic and non-epidemic settings for both data and samples, underscoring the necessity for investments in data standardisation, improved data storage and sharing infrastructure, and sample handling processes.

Notably, clear differences between PIs and non-PIs emerged in our findings. PIs more frequently highlighted increased authorship opportunities, cross-cohort collaborations with funding support, and short-term capacity building as motivators and benefits of sharing. These findings reflect their greater involvement in strategic decision-making and professional recognition opportunities, indicating potential disparities in career benefits and recognition within research teams. Economic and motivational barriers were also perceived to be more important by PIs than non-PIs, suggesting that the role of PIs in securing funding and ensuring recognition of research shapes their barrier perceptions. These differences underline the importance of explicitly addressing career and benefit-sharing disparities within research teams. Strategies to enhance data and sample sharing should therefore include clearly defined authorship guidelines for cohort members, equitable benefit-sharing mechanisms, and targeted career development opportunities for non-PIs. Such strategies can help build inclusive, collaborative environments, foster equitable participation in research, and ultimately enhance researcher willingness and ability to share data and samples in both epidemic and non-epidemic settings.

## Supplementary Information

Supplementary Material 1.

Supplementary Material 2.

## Data Availability

The survey and de-identified dataset of survey responses are available on OSF (https://doi.org/10.17605/OSF.IO/EDPSH).

## Abbreviations

AFI: Acute febrile illness
CHIKV: Chikungunya virus
Clin-epi: Clinical-epidemiological
DENV: Dengue virus
ERC: Ethics Review Committee
GDPR: General Data Protection Regulation
HIPPA: Health Insurance Portability and Accountability Act
HIV: Human immunodeficiency virus
IDAMS: International Research Consortium on Dengue Risk Assessment, Management and Surveillance
ID: Infectious disease
IDDO: Infectious Diseases Data Observatory
LMICs: Low-and-middle-income countries
MERS-CoV: Middle East Respiratory Syndrome Coronavirus
NTDs: Neglected tropical diseases
PEARL: Political, Ethical, Administrative, Regulatory, and Legal
PIs: Principal Investigators
WHO: World Health Organization
WWARN: World-Wide Malaria Research Network
ZIKV: Zika virus
