# Supplementary material for: Identifying the motivators, benefits and barriers to sharing participant-level data and samples: results from an international online survey of acute febrile illness cohort teams
Source: BMC Med Ethics. 2026 Feb 11;27:39. doi: 10.1186/s12910-026-01399-2 (PMC12930556; doi:10.1186/s12910-026-01399-2)
Supplement: Supplementary file 1 — Supplementary Material 1. [file 12910_2026_1399_MOESM1_ESM.pdf]

Data and sample sharing for acute febrile illness cohorts in low-and-middle-income countries

Dear Acute Febrile Illness Cohort researcher,

We would like to invite you to participate in the Data and sample sharing for acute febrile illness cohorts in low-and-middle-income countries survey.

The objective of this study is to quantify the relative importance of different barriers and facilitators to sharing de-identified, participant-level data and human biospecimens during and outside of the public health response to epidemics. Sharing de-identified, participant-level data and human biospecimens facilitates the development of new prevention strategies, treatments and prophylaxis. Survey findings may help funders and other stakeholders facilitate data and sample sharing during and outside of epidemic response.

Survey participants were identified through a review of existing lists of acute febrile illness-related cohorts in LMIC and through referral by research team members who participate in cross-cohort dengue and Zika virus initiatives.

We ask that cohort PI(s) complete the survey and forward the survey link survey to their laboratory and field data managers. Having multiple people from one team take the survey will allow us to understand whether members of the same team have different opinions about the most important barriers or facilitators to sharing participant-level data and human biospecimens.

We ask that cohorts based in high income countries or cohorts that focus on HIV, tuberculosis, or Ebola not participate in the survey as data and sample sharing within these groups may differ importantly from sharing in acute febrile illness cohorts that focus on other pathogens.

The online survey is administered through REDCap, a General Data Protection Regulation (GDPR)- and Health Insurance Portability and Accountability Act of 1996 (HIPAA)-compliant survey research platform. The survey should take about 15 minutes to complete.

Voluntary participation: Participation in the online survey is voluntary and participants are not required to answer any question. Participants will not receive financial compensation, nor any direct benefit, from participation.

Dissemination of results: Survey results will be made available through an Open Access publication and survey data will be made available for download at the Open Science Framework website.

Privacy policy: No data that could be used to identify individual studies will be described in the publication or included in the publicly available dataset. The name of the study PI and the location of the study are collected to ensure results can be clustered within given studies, but location will be replaced by a general categorization (e.g. Central America) and PI name by a number that uniquely identifies that given study.

Right to withdraw: Participants who wish to delete their data after fully or partially completing the study can contact the study Principal Investigator. Data will be deleted up until the publication of the survey results. Any request for deletion will necesitate a note that describes the general geographic location (e.g. Central America) and reason for requested deletion.

Ethical review: The survey research protocol was approved by the Heidelberg University Medical Faculty Ethics Review Committee (ERC# S-504/2020).

Funding information: The survey was developed as part of the RECODID project, funded by the EU Horizon 2020 research and innovation programme (grant agreement 825746) and the CIHR Institute of Genetics (grant agreement 01886-000).

Please direct any comments or questions to the survey principal investigator, Lauren Maxwell, PhD at: lauren.maxwell@uni-heidelberg.de.

By clicking on the forward button, you provide your consent to participate in the Data and sample sharing for acute febrile illness cohorts in low-and-middle-income countries online survey.

Languages:

- ◯ English
- ◯ Español
- ◯ Português

Respondent demographic information	
What is your primary affiliation?	◯ With the cohort itself<br>◯ Academic institution within the country where the cohort is based<br>◯ Academic institution outside of the country where the cohort is based<br>◯ Ministry of Health<br>◯ State or municipal-level health system<br>◯ Other
Please describe your primary affiliation:	__________________________________
What is your role within the cohort?	◯ Field data manager<br>◯ Laboratory data manager<br>◯ Statistician<br>◯ Field staff/data collector<br>◯ Laboratory scientist<br>◯ Principal Investigator<br>◯ Other
Please describe your role within the cohort:	____________________________
What is your age group?	◯ under 25<br>◯ 25-35<br>◯ 36-45<br>◯ 46-55<br>◯ over 55
How would you classify your career stage?	◯ early career (student, postdoc, < 5 years of work experience)<br>◯ mid career (5-15 years of experience)<br>◯ advanced career (PI, group leader, 16+ years of experience)
Are you from the country where the cohort is based?	◯ Yes<br>◯ No
Where are you from?	◯ Asia<br>◯ Europe<br>◯ Africa<br>◯ North or Central America<br>◯ South America<br>◯ Oceania

Which Central or North American country are you from?	◯ Antigua and Barbuda, AG<br>◯ Anguilla, AI<br>◯ Aruba, AW<br>◯ Bahamas, BS<br>◯ Barbados, BB<br>◯ Belize, BZ<br>◯ Bermuda, BM<br>◯ Bonaire, Sint Eustatius and Saba, BQ<br>◯ British Virgin Islands, VG<br>◯ Canada, CA<br>◯ Cayman Islands, KY<br>◯ Costa Rica, CR<br>◯ Cuba, CU<br>◯ Curaçao, CW<br>◯ Dominica, DM<br>◯ Dominican Republic, DO<br>◯ El Salvador, SV<br>◯ Greenland, GL<br>◯ Grenada, GD<br>◯ Guadeloupe, GP<br>◯ Guatemala, GT<br>◯ Haiti, HT<br>◯ Honduras, HN<br>◯ Jamaica, JM<br>◯ Martinique, MQ<br>◯ Mexico, MX<br>◯ Montserrat, MS<br>◯ Netherlands Antilles, AN<br>◯ Nicaragua, NI<br>◯ Panama, PA<br>◯ Puerto Rico, PR<br>◯ Saint Barthelemy. BL<br>◯ Saint Kitts and Nevis, KN<br>◯ Saint Lucia, LC<br>◯ Saint Martin (french part), MF<br>◯ Saint Pierre and Miquelon, PM<br>◯ Saint Vincent and the Grenadines, VC<br>◯ Saint Maarten (dutch part), SX<br>◯ Trinidad and Tobago, TT<br>◯ Turks and Caicos Islands, TC<br>◯ United States Minor Outlying Islands, UM<br>◯ United States of America, US<br>◯ United States Virgin Islands, VI
Which South American country are you from?	◯ Argentina, AR<br>◯ Bolivia, BO<br>◯ Brazil, BR<br>◯ Chile, CL<br>◯ Colombia, CO<br>◯ Ecuador, EC<br>◯ Falkland Islands (Malvinas), FK<br>◯ French Guiana, GF<br>◯ Guyana, GY<br>◯ Paraguay, PY<br>◯ Peru, PE<br>◯ Suriname, SR<br>◯ Uruguay, UY<br>◯ Venezuela, VE
Basic information about your cohort	
How many cohorts does your study team manage?	◯ one cohort<br>◯ more than one cohort

How are different cohorts defined?<br><br>Select all that apply	☐ year of birth<br>☐ pregnancy status<br>☐ pathogen<br>☐ geography<br>☐ source population (e.g. hospital, school, etc)<br>☐ other
In what other way are the different cohorts managed by your study team defined?	__________________________
For this survey, please consider any of your cohorts in your answers.	
What pathogen(s) or type of pathogen(s) does your febrile illness cohort focus on?<br><br>Select all that apply	☐ Dengue<br>☐ Zika<br>☐ Bacteria (other than M. tuberculosis)<br>☐ Fungi<br>☐ Protozoa (other than Plasmodium)<br>☐ SARS-CoV-2<br>☐ Other
What type of bacteria does your cohort focus on?	_____________________
What type of fungal pathogen does your cohort focus on?	_____________________
What type of protozoa does your cohort focus on?	_____________________
Please list the other pathogen(s) that your febrile illness cohort focuses on:	__________________________
What types of data or samples does your cohort currently collect or partner with outside groups to collect?<br><br>Please select all that apply	☐ Participant-level clinical-epidemiological data<br>☐ Human biological samples<br>☐ Pathogen biological samples<br>☐ Human genetic/OMICs data<br>☐ Pathogen genetic/OMICs data
Has your cohort ever collected human biological samples?	◯ Yes<br>◯ No
Has your cohort ever extracted human OMICs data from human biological samples?	◯ Yes<br>◯ No
At what level does your cohort recruit participants?	◯ Multi-country<br>◯ Country<br>◯ Multi-state/regional<br>◯ State<br>◯ Municipality<br>◯ City or village<br>◯ Individual school or hospital<br>◯ Other
At what other level does your cohort recruit participants?	_____________________

What is/are the last name(s) of the PI(s) of your cohort? __________________________________

Please note: this information will be used to understand the variation in responses from different people within one cohort. Cohort PIs will not be named in any publication.

Study location

Where is your cohort based?

Select all that apply

- ☐ Asia
- ☐ Europe
- ☐ Africa
- ☐ North or Central America
- ☐ South America
- ☐ Oceania
- ☐ Antarctica

In which African country or countries is your cohort based?

Select all that apply

- ☐ Algeria, DZ
- ☐ Angola, AO
- ☐ Benin, BJ
- ☐ Botswana, BW
- ☐ Burkina Faso, BF
- ☐ Burundi, BI
- ☐ Cabo Verde, CV
- ☐ Cameroon, CM
- ☐ Central African Republic, CF
- ☐ Chad, TD
- ☐ Comoros, KM
- ☐ Cote d'Ivoire, CI
- ☐ Democratic Republic of the Congo (DRC), CD
- ☐ Djibouti, DJ
- ☐ Egypt, EG
- ☐ Equatorial Guinea, GQ
- ☐ Eritrea, ER
- ☐ Eswatini, SZ
- ☐ Ethiopia, ET
- ☐ Gabon, GA
- ☐ Gambia, GM
- ☐ Ghana, GH
- ☐ Guinea-Bissau, GW
- ☐ Guinea, GN
- ☐ Kenya, KE
- ☐ Lesotho, LS
- ☐ Liberia, LR
- ☐ Libya, LY
- ☐ Madagascar, MG
- ☐ Malawi, MW
- ☐ Mali, ML
- ☐ Mauritania, MR
- ☐ Mauritius, MU
- ☐ Mayotte, YT
- ☐ Morocco, MA
- ☐ Mozambique, MZ
- ☐ Namibia, NA
- ☐ Niger, NE
- ☐ Nigeria, NG
- ☐ Republic of the Congo (Congo-Brazzaville), CG
- ☐ Reunion, RE
- ☐ Rwanda, RW
- ☐ Saint Helena, Ascension and Tristan da Cunha, SH
- ☐ Sao Tome and Principe, ST
- ☐ Senegal, SN
- ☐ Seychelles, SC
- ☐ Sierra Leone, SL
- ☐ Somalia, SO
- ☐ South Africa, ZA
- ☐ South Sudan, SS
- ☐ Sudan, SD
- ☐ Tanzania, TZ
- ☐ Togo, TG
- ☐ Tunisia, TN
- ☐ Uganda, UG
- ☐ Western Sahara, EH
- ☐ Zambia, ZM
- ☐ Zimbabwe, ZW

In which Asian country or countries is your cohort based?

Select all that apply

- ☐ Afghanistan, AF
- ☐ Armenia, AM
- ☐ Azerbaijan, AZ
- ☐ Bahrain, BH
- ☐ Bangladesh, BD
- ☐ Bhutan, BT
- ☐ British Indian Ocean Territory (Chagos Archipelago), IO
- ☐ Brunei, BN
- ☐ Cambodia, KH
- ☐ China, CN
- ☐ Christmas Island, CX
- ☐ Cocos (Keeling) Islands, CC
- ☐ Cyprus, CY
- ☐ Georgia, GE
- ☐ Hong Kong, Special Administrative Region of China, HK
- ☐ India, IN
- ☐ Indonesia, ID
- ☐ Iran, IR
- ☐ Iraq, IQ
- ☐ Iraq-Saudi Arabia Neutral Zone
- ☐ Israel, IL
- ☐ Japan, JP
- ☐ Jordan, JO
- ☐ Kazakhstan, KZ
- ☐ Kuwait, KW
- ☐ Kyrgyz Republic, KG
- ☐ Lao People's Democratic Republic, LA
- ☐ Lebanon, LB
- ☐ Macao, Special Administrative Region of China, MO
- ☐ Malaysia, MY
- ☐ Maldives, MV
- ☐ Mongolia, MN
- ☐ Myanmar, MM
- ☐ Nepal, NP
- ☐ North Korea, KP
- ☐ Oman, OM
- ☐ Pakistan, PK
- ☐ Palestinian Territory, PS
- ☐ Philippines, PH
- ☐ Qatar, QA
- ☐ Russian Federation, RU
- ☐ Saudi Arabia, SA
- ☐ Singapore, SG
- ☐ South Korea, KR
- ☐ Spratly Islands
- ☐ Sri Lanka, LK
- ☐ Syrian Arab Republic, SY
- ☐ Taiwan, TW
- ☐ Tajikistan, TJ
- ☐ Thailand, TH
- ☐ Timor-Leste, TL
- ☐ Turkey, TR
- ☐ Turkmenistan, TM
- ☐ United Arab Emirates, AE
- ☐ United Nations Neutral Zone
- ☐ Uzbekistan, UZ
- ☐ Vietnam, VN
- ☐ Yemen, YE

In which North or Central American country or countries is your cohort based?

Select all that apply

- ☐ Antigua and Barbuda, AG
- ☐ Anguilla, AI
- ☐ Aruba, AW
- ☐ Bahamas, BS
- ☐ Barbados, BB
- ☐ Belize, BZ
- ☐ Bermuda, BM
- ☐ Bonaire, Sint Eustatius and Saba, BQ
- ☐ British Virgin Islands, VG
- ☐ Canada, CA
- ☐ Cayman Islands, KY
- ☐ Costa Rica, CR
- ☐ Cuba, CU
- ☐ Curaçao, CW
- ☐ Dominica, DM
- ☐ Dominican Republic, DO
- ☐ El Salvador, SV
- ☐ Greenland, GL
- ☐ Grenada, GD
- ☐ Guadeloupe, GP
- ☐ Guatemala, GT
- ☐ Haiti, HT
- ☐ Honduras, HN
- ☐ Jamaica, JM
- ☐ Martinique, MQ
- ☐ Mexico, MX
- ☐ Montserrat, MS
- ☐ Netherlands Antilles, AN
- ☐ Nicaragua, NI
- ☐ Panama, PA
- ☐ Puerto Rico, PR
- ☐ Saint Barthelemy. BL
- ☐ Saint Kitts and Nevis, KN
- ☐ Saint Lucia, LC
- ☐ Saint Martin (french part), MF
- ☐ Saint Pierre and Miquelon, PM
- ☐ Saint Vincent and the Grenadines, VC
- ☐ Saint Maarten (dutch part), SX
- ☐ Trinidad and Tobago, TT
- ☐ Turks and Caicos Islands, TC
- ☐ United States Minor Outlying Islands, UM
- ☐ United States of America, US
- ☐ United States Virgin Islands, VI

In which South American country or countries is your cohort based?

Select all that apply

- ☐ Argentina, AR
- ☐ Bolivia, BO
- ☐ Brazil, BR
- ☐ Chile, CL
- ☐ Colombia, CO
- ☐ Ecuador, EC
- ☐ Falkland Islands (Malvinas), FK
- ☐ French Guiana, GF
- ☐ Guyana, GY
- ☐ Paraguay, PY
- ☐ Peru, PE
- ☐ Suriname, SR
- ☐ Uruguay, UY
- ☐ Venezuela, VE

In which state is your cohort based?

____

What is the age range for your cohort's participants?	__________________________________
What is the source population for your cohort?<br><br>Select all that apply	☐ Community based<br>☐ Hospital or health center based<br>☐ School based<br>☐ Other
What is the other source population for your cohort?	____
Other than age criteria, does your cohort limit recruitment to any of the following groups?<br><br>Select all that apply	☐ Pregnant women<br>☐ Women of reproductive age (pregnant or not)<br>☐ Minors (age 18 and under)<br>☐ Other
Which other specially defined group is recruitment into your cohort limited to?	____
Sharing de-identified, participant-level data	
What types of data or samples does your cohort currently share?<br><br>Select all that apply	☐ Participant-level clinical-epidemiological data<br>☐ Human biological samples<br>☐ Pathogen biological samples<br>☐ Human genetic/OMICs data<br>☐ Pathogen genetic/OMICs data<br>☐ Cohort does not currently share data or samples
With which groups does your cohort currently share de-identified participant-level data?<br><br>Please select all that apply	☐ Other cohorts within a consortium (e.g. DENCO, ZIKAlliance)<br>☐ Other cohort(s), not within a consortium<br>☐ State or city-level Health Department<br>☐ National Ministry of Health<br>☐ Academic institution, not otherwise engaged in cohort<br>☐ Private industry, not otherwise engaged in cohort<br>☐ Non-academic, non-profit industry, not otherwise engaged in cohort<br>☐ Other
With which consortium/consortia does your cohort currently share de-identified, participant-level data?	____
With which other group(s) does your cohort currently share de-identified, participant-level data?	____
Has your cohort ever shared de-identified, participant-level data?	◯ Yes<br>◯ No
When did your cohort share de-identified, participant-level data?<br><br>Please select all that apply if multiple-instances of data sharing	☐ As soon as it was collected (real time)<br>☐ < = 1 year after data collection<br>☐ > 1 year after data collection<br>☐ After publication of main findings<br>☐ Other

Please describe when your cohort shared de-identified, participant-level data:	__________________________
When your cohort shared de-identified participant-level data in real time was it during an epidemic?	◯ Yes, during an epidemic<br>◯ No, in a non-epidemic setting<br>◯ In both an epidemic and non-epidemic setting
Is your cohort sharing data in real time during the COVID-19 pandemic?	◯ Yes, this is the first time we share data in real time<br>◯ Yes, but this is not the first time we share data in real time<br>◯ No
Sharing human biological samples	
With which groups does your cohort currently share human biological samples?<br><br>Please select all that apply	☐ Other cohorts within a consortium (e.g. DENCO, ZIKAlliance)<br>☐ Other cohort(s) NOT within a consortium<br>☐ Local health department (state/department or regional level)<br>☐ National health system (e.g., MoH)<br>☐ Academic institution, not otherwise engaged in cohort<br>☐ Private industry, not otherwise engaged in cohort<br>☐ Non-academic, non-profit industry, not otherwise engaged in cohort<br>☐ Other
With which consortium/consortia does your cohort currently share human biological samples?	__________________________
With which other group(s) does your cohort currently share human biological sample data?	__________________________
Has your cohort ever shared human biological samples?	◯ Yes<br>◯ No
When did your cohort share the human biological samples?<br><br>Please select all that apply if multiple-instances of sample sharing	☐ Soon after collection (~real time)<br>☐ < = 1 year after sample collection<br>☐ > 1 year after sample collection<br>☐ After publication of main findings<br>☐ Other
Please describe when your cohort shared human biological samples:	__________________________
When your cohort shared human biological samples in real time was it during an epidemic?	◯ Yes, during an epidemic<br>◯ No, in a non-epidemic setting<br>◯ In both an epidemic and non-epidemic setting

Is your cohort sharing human biological samples in real time during the COVID-19 pandemic?	◯ Yes, this is the first time we share human biological samples in real time<br>◯ Yes, but this is not the first time we share human biological samples in real time<br>◯ No
Sharing human OMICs data	
With which groups does your cohort currently share human OMICs data?<br><br>Please select all that apply	☐ Other cohorts within a consortium (e.g. DENCO, ZIKAlliance)<br>☐ Other cohort(s) NOT within a consortium<br>☐ Local health department (state/department or regional level)<br>☐ National health system (e.g., MoH)<br>☐ Academic institution, not otherwise engaged in cohort<br>☐ Private industry, not otherwise engaged in cohort<br>☐ Non-academic, non-profit industry, not otherwise engaged in cohort<br>☐ Other
With which consortium/consortia does your cohort currently share human OMICs data?	__________________________________
With which other group(s) does your cohort currently share human OMICs data?	__________________________________
Has your cohort ever shared human OMICs data?	◯ Yes<br>◯ No
When did your cohort share the human OMICs data?<br><br>Please select all that apply if multiple-instances of sample sharing	☐ Soon after collection (real time)<br>☐ < = 1 year after sample collection<br>☐ > 1 year after sample collection<br>☐ After publication of main findings<br>☐ Other
Please describe when your cohort shared human OMICs data:	__________________________________
When your cohort shared human oMICs data in real time was it during an epidemic?	◯ Yes, during an epidemic<br>◯ No, in a non-epidemic setting<br>◯ In both an epidemic and non-epidemic setting
Is your cohort sharing OMICs data in real time during the COVID-19 pandemic?	◯ Yes, this is the first time we share OMICs data in real time<br>◯ Yes, but this is not the first time we share OMICs data in real time<br>◯ No
Best and worst experiences with data or sample sharing	

Please describe your WORST experience sharing de-identified, participant-level clinical or human OMICs data or human biospecimens from your cohort? __________________________________

Which group or type of group did you share with?

What made that your worse data or sample sharing experience?

Please describe your BEST experience sharing de-identified, participant-level clinical or human OMICs data or human biospecimens from your cohort __________________________________

Which group or type of group did you share with?

What made that your best data or sample sharing experience?

Motivation for data and sample sharing

What do you see as the most important MOTIVATION for your cohort to share the following data types with cohorts with which you do not have an established partnership (e.g. shared grant)

---

deidentified, participant-level data

Select up to three choices

- ☐ Funder requirement
- ☐ Local health department (state/department or regional level) requirement
- ☐ National MoH requirement
- ☐ Cross-cohort or international study with funding support
- ☐ Cross-cohort or international study without funding support (e.g. individual participant data meta-analysis)
- ☐ Development of novel vaccines, treatments, or therapies
- ☐ Public health rationale, other than development of novel vaccines, treatments, or therapies
- ☐ Prevent duplication of efforts
- ☐ Inform future research investments
- ☐ Increased funding opportunities or opportunities for collaboration
- ☐ Increased authorship opportunities
- ☐ Other
- ☐ There is no motivation for sharing de-identified clinical data

---

What other important MOTIVATION is there for your cohort to share de-identified, participant-level data? __________________________________

human biological samples

Select up to three choices

- ☐ Funder requirement
- ☐ Local health department (state/department or regional level) requirement
- ☐ National MoH requirement
- ☐ Cross-cohort or international study with funding support
- ☐ Cross-cohort or international study without funding support (e.g. individual participant data meta-analysis)
- ☐ Development of novel vaccines, treatments, or therapies
- ☐ Public health rationale, other than development of novel vaccines, treatments, or therapies
- ☐ Prevent duplication of efforts
- ☐ Inform future research investments
- ☐ Increased funding opportunities or opportunities for collaboration
- ☐ Increased authorship opportunities
- ☐ Other
- ☐ There is no motivation for sharing human biological samples

What other important MOTIVATION is there for your cohort to share human biological samples?

____

human genetic/OMICs data

Select up to three choices

- ☐ Funder requirement
- ☐ Local health department (state/department or regional level) requirement
- ☐ National MoH requirement
- ☐ Cross-cohort or international study with funding support
- ☐ Cross-cohort or international study without funding support (e.g. individual participant data meta-analysis)
- ☐ Development of novel vaccines, treatments, or therapies
- ☐ Public health rationale, other than development of novel vaccines, treatments, or therapies
- ☐ Prevent duplication of efforts
- ☐ Inform future research investments
- ☐ Increased funding opportunities
- ☐ Increased authorship opportunities
- ☐ Other
- ☐ There is no motivation for sharing human OMICs data

What other important MOTIVATION is there for your cohort to share human genetic/OMICs data?

____

Barriers to sharing participant-level data during an epidemic

How important are the following barriers to sharing de-identified participant-level data with cohorts with which you did not have a pre-established partnership DURING AN EPIDEMIC?

Technical

lack of shared data dictionary difficult to harmonize data accross studies because variables do not map to an international standard (e.g. C-DASH, SNOMED) outside groups can misinterpret data or analyze the data in a way that leads to inaccurate conclusions

Not very important | Somewhat important | Very important

(Place a mark on the scale above)

Motivational

external groups will be able to publish results faster than team that collected data research team needs to use data to generate publications and additional funding data generators may not receive credit for their work data sharing can affect research participants' trust in the project lack of public health rationale

Not very important | Somewhat important | Very important

(Place a mark on the scale above)

Economic - Resource-related

lack of funding for time needed to curate data for sharing, annote dataset, or answer questions about dataset

Not very important | Somewhat important | Very important

(Place a mark on the scale above)

Regulatory

informed consent forms do not allow for data sharing (no future use) lack of permission from ethics review committee or institutional review board not clear who "owns" the data or samples lack of permission from funder

Not very important | Somewhat important | Very important

(Place a mark on the scale above)

Legal

restrictive agreements with collaborators lack of clear guidance from MOH concerns about sharing with countries with different legal frameworks or different interpretations of International Health Regulations (IHR)

Not very important | Somewhat important | Very important

(Place a mark on the scale above)

Ethical

risks associated with sharing data outweighs benefit to source population need to protect patient privacy no or limited benefits to sharing for cohort staff external groups may use data for financial gain (patents, vaccines, diagnostics)

Not very important | Somewhat important | Very important

(Place a mark on the scale above)

Political

Ministry of Health opposes sharing data Local health authorities do not publish or allow for the dissemination of data

Not very important — Somewhat important — Very important

(Place a mark on the scale above)

In addition to the barriers mentioned here, please describe any additional barriers to sharing de-identified participant-level data in an epidemic setting:

____

Barriers to sharing de-identified, participant-level data outside of an epidemic setting

How important are the following barriers to sharing de-identified participant-level data with cohorts with which you did not have a pre-established partnership OUTSIDE OF AN EPIDEMIC SETTING?

Technical

lack of shared data dictionary difficult to harmonize data accross studies because variables do not map to an international standard (e.g. C-DASH, SNOMED) outside groups can misinterpret data or analyze the data in a way that leads to inaccurate conclusions

Not very important — Somewhat important — Very important

(Place a mark on the scale above)

Motivational

external groups will be able to publish results faster than team that collected data research team needs to use data to generate publications and additional funding data generators may not receive credit for their work data sharing can affect research participants' trust in the project lack of public health rationale

Not very important — Somewhat important — Very important

(Place a mark on the scale above)

Economic - Resource-related

lack of funding for time needed to curate data for sharing, annote dataset, or answer questions about dataset

Not very important — Somewhat important — Very important

(Place a mark on the scale above)

Regulatory

informed consent forms do not allow for data sharing (no future use) lack of permission from ethics review committee or institutional review board not clear who "owns" the data or samples lack of permission from funder

Not very important — Somewhat important — Very important

(Place a mark on the scale above)

Legal

restrictive agreements with collaborators lack of clear guidance from MOH concerns about sharing with countries with different legal frameworks or different interpretations of International Health Regulations (IHR)

Not very important | Somewhat important | Very important

(Place a mark on the scale above)

Ethical

risks associated with sharing data outweighs benefit to source population need to protect patient privacy no or limited benefits to sharing for cohort staff external groups may use data for financial gain (patents, vaccines, diagnostics)

Not very important | Somewhat important | Very important

(Place a mark on the scale above)

Political

Ministry of Health opposes sharing data Local health authorities do not publish or allow for the dissemination of data

Not very important | Somewhat important | Very important

(Place a mark on the scale above)

In addition to the barriers mentioned here, please describe any additional barriers to sharing de-identified participant-level data in a non-epidemic setting:

____

Barriers to sharing human biological samples during an epidemic

How important are the following barriers to sharing human biological samples with cohorts with which you did not have a pre-established partnership DURING AN EPIDEMIC?

Technical

Additional paperwork associated with sample transfer Need for material transfer agreements No metadata standards accross biorepositories Difficult to track chain of custody with external sample transfers

Not very important | Somewhat important | Very important

(Place a mark on the scale above)

Motivational

external groups will be able use samples to develop new research or interventions faster than team that collected the samples research team needs to use the samples to generate publications and secure additional funding cohort team may not receive credit for their work sample sharing can affect research participants' trust in the project lack of public health rationale

Not very important | Somewhat important | Very important

(Place a mark on the scale above)

Economic - Resource-related

lack of funding for time needed to organize the sample catalogue for sharing, prepare samples for shipment, or connect participant-level data to sample data to accurately characterize samples limited sample volume

Not very important | Somewhat important | Very important

(Place a mark on the scale above)

Regulatory

informed consent forms do not allow for sample sharing (no future use) lack of permission from ethics review committee or institutional review board not clear who "owns" the samples lack of permission from funder

Not very important | Somewhat important | Very important

(Place a mark on the scale above)

Legal

restrictive agreements with collaborators lack of clear guidance from MOH concerns about sharing with countries with different legal frameworks or different interpretations of International Health Regulations (IHR)

Not very important | Somewhat important | Very important

(Place a mark on the scale above)

Ethical

risks associated with sharing samples outweighs benefit to source population need to protect patient privacy no or limited benefits to sharing for cohort staff external groups may use samples for financial gain (patents, vaccines, diagnostics)

Not very important | Somewhat important | Very important

(Place a mark on the scale above)

Political

Ministry of Health opposes sample sharing Local health authorities do not allow for sample sharing

Not very important | Somewhat important | Very important

(Place a mark on the scale above)

In addition to the barriers mentioned here, please describe any additional barriers to sharing human biological samples in an epidemic setting: __________________________________

Barriers to sharing human biological samples outside of an epidemic setting

How important are the following barriers to sharing human samples with cohorts with which you did not have a pre-established partnership OUTSIDE OF AN EPIDEMIC SETTING?

Technical

Additional paperwork associated with sample transfer Need for material transfer agreements No metadata standards accross biorepositories Difficult to track chain of custody with external sample transfers

Not very important | Somewhat important | Very important

(Place a mark on the scale above)

Motivational

external groups will be able use samples to develop new research or interventions faster than team that collected the samples research team needs to use the samples to generate publications and secure additional funding cohort team may not receive credit for their work sample sharing can affect research participants' trust in the project lack of public health rationale

Not very important | Somewhat important | Very important

(Place a mark on the scale above)

Economic - Resource-related

lack of funding for time needed to organize the sample catalogue for sharing, prepare samples for shipment, or connect participant-level data to sample data to accurately characterize samples limited sample volume

Not very important | Somewhat important | Very important

(Place a mark on the scale above)

Regulatory

informed consent forms do not allow for sample sharing (no future use) lack of permission from ethics review committee or institutional review board not clear who "owns" the samples lack of permission from funder

Not very important | Somewhat important | Very important

(Place a mark on the scale above)

Legal

restrictive agreements with collaborators lack of clear guidance from MOH concerns about sharing with countries with different legal frameworks or different interpretations of International Health Regulations (IHR)

Not very important | Somewhat important | Very important

(Place a mark on the scale above)

Ethical

risks associated with sharing samples outweighs benefit to source population need to protect patient privacy no or limited benefits to sharing for cohort staff external groups may use samples for financial gain (patents, vaccines, diagnostics)

Not very important | Somewhat important | Very important

(Place a mark on the scale above)

Political

Ministry of Health opposes sample sharing Local health authorities do not allow for sample sharing

Not very important | Somewhat important | Very important

(Place a mark on the scale above)

In addition to the barriers mentioned here, please describe any additional barriers to sharing human biological samples in a non epidemic setting: __________________________________

Barriers to sharing human OMICs data during an epidemic

How important are the following barriers to sharing human OMICs data with cohorts with which you did not have a pre-established partnership DURING AN EPIDEMIC?

Technical

lack of shared metadata dictionary difficult to harmonize data accross studies because of different standards for OMICs metadata outside groups can misinterpret data or analyze the data in a way that leads to inaccurate conclusions

Not very important | Somewhat important | Very important

(Place a mark on the scale above)

Motivational

external groups will be able to publish results faster than team that collected data research team needs to use OMICs data to generate publications and additional funding data generators may not receive credit for their work data sharing can affect research participants' trust in the project lack of public health rationale

Not very important | Somewhat important | Very important

(Place a mark on the scale above)

Economic - Resource-related

lack of funding for time needed to curate OMICs data for sharing, annote dataset, or answer questions about dataset

Not very important | Somewhat important | Very important

(Place a mark on the scale above)

Regulatory

informed consent forms do not allow for human OMICs data sharing (no future use) lack of permission from ethics review committee or institutional review board not clear who "owns" the human OMICs data lack of permission from funder

Not very important | Somewhat important | Very important

(Place a mark on the scale above)

Legal

restrictive agreements with collaborators lack of clear guidance from MOH concerns about sharing OMICs data with countries with different legal frameworks or different interpretations of International Health Regulations (IHR)

Not very important | Somewhat important | Very important

(Place a mark on the scale above)

Ethical

risks associated with sharing data outweighs benefit to source population need to protect patient privacy no or limited benefits to sharing for cohort staff external groups may use OMICs data for financial gain (patents, vaccines, diagnostics)

Not very important | Somewhat important | Very important

(Place a mark on the scale above)

Political

Ministry of Health opposes sharing human OMICs data Local health authorities do not publish or allow for the dissemination of human OMICs data

Not very important | Somewhat important | Very important

(Place a mark on the scale above)

In addition to the barriers mentioned here, please describe any additional barriers to sharing human OMICs data in an epidemic setting:

____

Barriers to sharing human OMICs outside of an epidemic setting

How important are the following barriers to sharing human OMICs data with cohorts with which you did not have a pre-established partnership OUTSIDE OF AN EPIDEMIC SETTING?

Technical

lack of shared metadata dictionary difficult to harmonize data accross studies because of different standards for OMICs metadata outside groups can misinterpret data or analyze the data in a way that leads to inaccurate conclusions

Not very important | Somewhat important | Very important

(Place a mark on the scale above)

Motivational

external groups will be able to publish results faster than team that collected data research team needs to use OMICs data to generate publications and additional funding data generators may not receive credit for their work data sharing can affect research participants' trust in the project lack of public health rationale

Not very important | Somewhat important | Very important

(Place a mark on the scale above)

Economic - Resource-related

lack of funding for time needed to curate OMICs data for sharing, annote dataset, or answer questions about dataset

Not very important | Somewhat important | Very important

(Place a mark on the scale above)

Regulatory

informed consent forms do not allow for human OMICs data sharing (no future use) lack of permission from ethics review committee or institutional review board not clear who "owns" the human OMICs data lack of permission from funder

Not very important | Somewhat important | Very important

(Place a mark on the scale above)

Legal

restrictive agreements with collaborators lack of clear guidance from MOH concerns about sharing OMICs data with countries with different legal frameworks or different interpretations of International Health Regulations (IHR)

Not very important | Somewhat important | Very important

(Place a mark on the scale above)

Ethical

risks associated with sharing data outweighs benefit to source population need to protect patient privacy no or limited benefits to sharing for cohort staff external groups may use OMICs data for financial gain (patents, vaccines, diagnostics)

Not very important | Somewhat important | Very important

(Place a mark on the scale above)

Political

Ministry of Health opposes sharing human OMICs data Local health authorities do not publish or allow for the dissemination of human OMICs data

Not very important | Somewhat important | Very important

(Place a mark on the scale above)

In addition to the barriers mentioned here, please describe any additional barriers to sharing human OMICs data in a non-epidemic setting: ____________________

SARS-CoV-2 Data and Sample Sharing

Has or will your site/study input/upload de-identified participant-level clinical-epidemiological data into/to any of the following COVID-19-related data sharing platforms or repositories?<br><br>Select all that apply	☐ ISARIC COVID-19 Data Platform<br>☐ SARS-CoV-2 EMBL Data Hubs<br>☐ American Society of Clinical Oncology (ASCO) COVID-19 in Oncology Registry<br>☐ CAPACITY COVID registry<br>☐ COVID-19 CVD Registry<br>☐ COVID-HEP Registry<br>☐ data.world<br>☐ Dermatology COVID-19 registry<br>☐ Discovery VIRUS COVID-19 Registry<br>☐ Extracorporeal Life Support Organization (ELSO) Registry<br>☐ GitHub<br>☐ HIV and COVID-19 Registry<br>☐ I-COVID (Italian Registry on Coronavirus in Pregnancy)<br>☐ Obvio-19<br>☐ PRIORITY (Pregnancy CoRonavIrus Outcomes RegIsTrY)<br>☐ QMENTA (MS Global Data-Sharing Initiative)<br>☐ Rheumatology COVID-19 Registry<br>☐ SECURE-Cirrhosis<br>☐ Surveillance Epidemiology of Coronavirus Under Research Exclusion (SECURE-IBD)<br>☐ Other
What other data sharing platform or repository has or will your study/site share COVID-19-related data to?	__________________________________
Broad consent for data or sample sharing	
Does the informed consent form for participation in your cohort include a provision for any of the following:	
use of participant data for research purposes other than those specified in the current study<br><br>(i.e. broad consent for future use of de-identified participant-level data)	○ Yes ○ No ○ Unsure
use of human biological samples for research purposes other than those specified in the current study<br><br>(i.e. broad consent for future use of human biological samples)	○ Yes ○ No ○ Unsure
extracting genetic data from human samples collected in the current study<br><br>(i.e. future use or broad consent for future use of genetic data derived from human samples)	○ Yes ○ No ○ Unsure
Data preservation, retrieval, and sharing systems	
Does your study use paper forms or data collection software (e.g. REDCap, Google Docs, KoboToolbox) for data collection?	○ paper forms only<br>○ electronic data capture software only<br>○ both paper forms and electronic data capture software

Do the variables in your data dictionary directly correspond to any of the following internationally recognized standards?<br><br>Please select all that apply	☐ ICD-9, 10, or 11<br>☐ SDTM<br>☐ CDASH<br>☐ SNOMED CT<br>☐ LOINC<br>☐ NCI<br>☐ CPT4<br>☐ RxNORM<br>☐ CVX<br>☐ UCUM<br>☐ NDF<br>☐ MedDRA<br>☐ HL7<br>☐ Other<br>☐ Variables do not directly correspond to an internationally recognized standard
Which other internationally recognized standard(s) do the variables in your data dictionary map to?	__________________________________
Does your cohort use any of the following biobanking platforms?<br><br>Please select all that apply	☐ LDMS<br>☐ MBioLIMS<br>☐ BioBank (TECHNIDATA)<br>☐ CloudLIMS<br>☐ Genohm SLIMS<br>☐ OpenSpecimen<br>☐ BSI (Biological Specimen Inventory)<br>☐ BiobankPro (Brooks)<br>☐ Labvantage 8<br>☐ STARLIMS<br>☐ Gemini Matrix<br>☐ TD-Biobank<br>☐ Freezerworks<br>☐ Bika LIMS<br>☐ Ark Informatics<br>☐ eB3Kit Biobank in a Box (Bibbox)<br>☐ In-house system<br>☐ Other<br>☐ Unsure
Which other platform does your cohort use for biobanking?	__________________________________

Does your cohort share human OMICs data on any of the following platforms?<br><br>Select all that apply	☐ CNGBdb (China National GeneBank DataBase)<br>☐ COMPARE<br>☐ DDBJ (Data Bank of Japan)<br>☐ EGA (European Genome-Phenome Archive)<br>☐ ELIXIR<br>☐ ENA (European Nucleotide Archive)<br>☐ GenBank<br>☐ GWH (Genome Warehouse)<br>☐ GISAID (Global Initiative on Sharing All Influenza Data)<br>☐ Lifebit<br>☐ NextStrain<br>☐ NMDC (National Microbiome Data Collaborative)<br>☐ Seven Bridges Genomics<br>☐ Other ELIXIR core data resources<br>ixir-europe.org/platforms/data/core-data-resources)<br>☐ Other
What other platforms does your study use to share human OMICS data?	__________________________________

For each datatype listed below, does your cohort have a clearly documented process for external groups to access that datatype which includes specific criteria for how applications are evaluated?

De-identified participant-level data	○ Yes<br>○ No<br>○ Unsure<br>○ We do not share de-identified participant-level data with external groups
Human biological samples	○ Yes<br>○ No<br>○ Unsure<br>○ We do not share human biological samples with external groups
Human OMICS data	○ Yes<br>○ No<br>○ Unsure<br>○ We do not share human OMICs data with external groups

Does your cohort currently participate in any of the following cross-cohort data or sample sharing initiatives?

cloud-based repository for cross-cohort participant-level data sharing	○ Yes ○ No ○ Unsure

shared biobank/biorepository that includes samples from more than one cohort	◯ Yes ◯ No ◯ Unsure
cross-cohort consortium wherein data or samples are shared between consortium partners without the use of a cloud-based system for de-centralized data sharing or a biobanking platform	◯ Yes ◯ No ◯ Unsure

Benefit sharing is one way for researchers to try to ensure that the community and/or the cohort staff receive some benefit from data or sample sharing.

Individual cohorts may not receive a clear benefit from data or sample sharing. Considering the effort needed to share data or samples with non-for-profit groups external to your project or consortium, what are the most important ways for your cohort to benefit from:

Sharing participant-level clinical-epidemiological data<br><br>Please select all that apply	☐ Increased opportunities for authorship<br>☐ Increased funding opportunities<br>☐ Reduced duplication of efforts<br>☐ Enhanced insights through collaboration<br>☐ Free or reduced cost access to novel diagnostics, treatments, or prophylaxis<br>☐ Short term capacity building investment (e.g. short course)<br>☐ Long-term capacity building investment (e.g. funded postdoc)<br>☐ Other<br>☐ There is no way that our cohort could benefit from sharing participant-level data
What is the most important way for your cohort to benefit from sharing participant-level clinical-epidemiological data?	__________________________________
Sharing human biological samples<br><br>Please select all that apply	☐ Access fee<br>☐ Joint ownership of rights to IP produced using samples<br>☐ Expanded grant opportunities<br>☐ Short term capacity building investment (e.g. short course)<br>☐ Long-term capacity building investment (e.g. funded postdoc)<br>☐ Participation in a multi-site biobanking network<br>☐ Infrastructure funding for biorepository<br>☐ Free or reduced cost access to novel diagnostics, treatments, or prophylaxis<br>☐ Other<br>☐ There is no way that our cohort could benefit from sharing participant biospecimens<br>☐ Not applicable, our cohort does not collect human samples
What is the most important way for your cohort to benefit from sharing human biological samples?	__________________________________

Sharing human OMICs data

Please select all that apply

- ☐ Increased opportunities for authorship
- ☐ Increased funding opportunities
- ☐ Reduced duplication of efforts
- ☐ Enhanced insights through collaboration
- ☐ Free or reduced cost access to novel diagnostics, treatments, or prophylaxis
- ☐ Short term capacity building investment (e.g. short course)
- ☐ Long-term capacity building investment (e.g. funded postdoc)
- ☐ Other
- ☐ There is no way that our cohort could benefit from sharing participant-level data

---

What is the most important way for your cohort to benefit from sharing human OMICs data?

____

Final thoughts

What is the most difficult data type to share in real time during an epidemic?

- ◯ clinical-epidemiological data
- ◯ human biological samples
- ◯ human OMICs data

What step could funders or researchers take to facilitate real time sharing of [epidemicshare_mostdiff] during a pandemic?

____

How has the COVID-19 pandemic changed your attitude toward data or sample sharing?

____

What would you like funders to know about sharing de-identified clinical-epidemiological data or human samples?

____

Please add anything else you would like to share related to data or sample sharing in acute febrile illness cohorts here:

____

Our research team has developed a COVID-19-specific metadata and data and sample sharing survey. Please consider completing the survey, here: https://cru.med.uni-heidelberg.de/redcap/surveys/?s=F74NKNC7AP.

Surveytext
