# Supplementary material for: Identifying the motivators, benefits and barriers to sharing participant-level data and samples: results from an international online survey of acute febrile illness cohort teams
Source: BMC Med Ethics. 2026 Feb 11;27:39. doi: 10.1186/s12910-026-01399-2 (PMC12930556; doi:10.1186/s12910-026-01399-2)
Supplement: Supplementary file 2 — Supplementary Material 2. [file 12910_2026_1399_MOESM2_ESM.docx]

# **Appendix File for:** Understanding the motivators and barriers to sharing participant-level data and samples: A cross-sectional study with acute febrile illness cohort teams

**Appendix Figure 1A. Comparison of motivators for sharing participant-level clinical-epidemiological data by respondent role in the cohort (PI vs non-PI)**


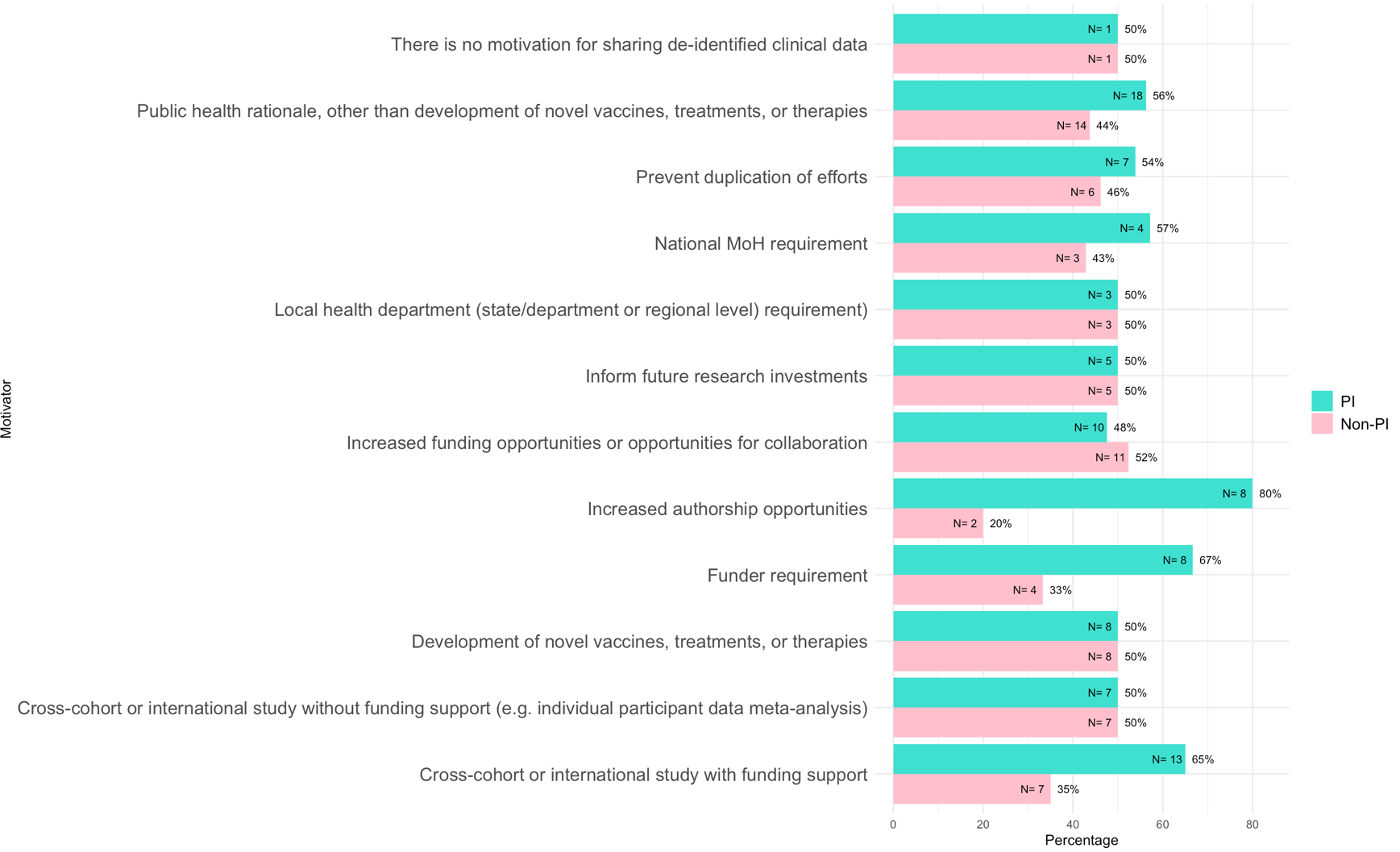


**Appendix Figure 1B. Comparison of motivators for sharing human biological samples by respondent role in the cohort (PI vs non-PI)**


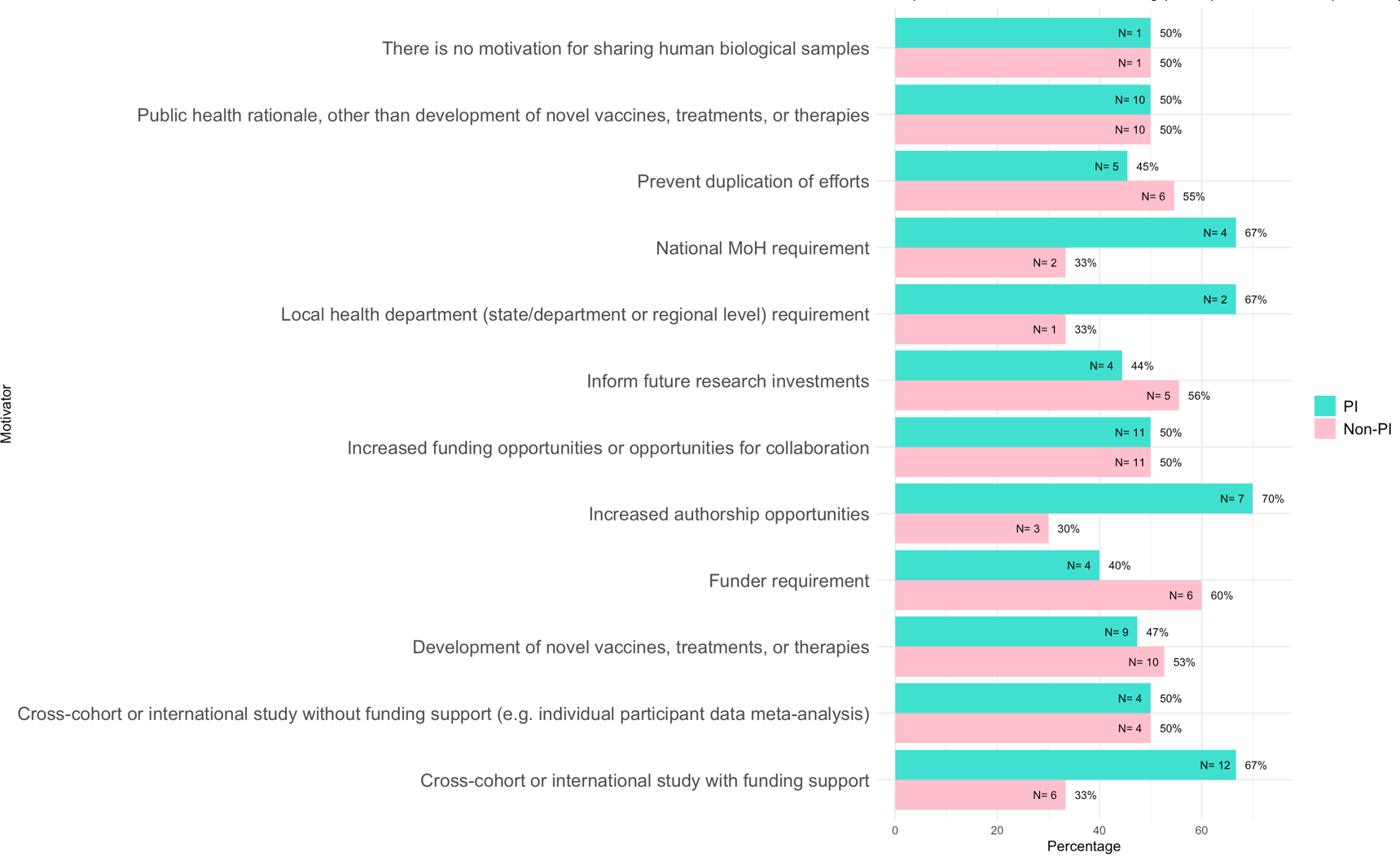


**Appendix Figure 1C. Comparison of motivators for sharing human genetic data by respondent role in the cohort (PI vs non-PI)**


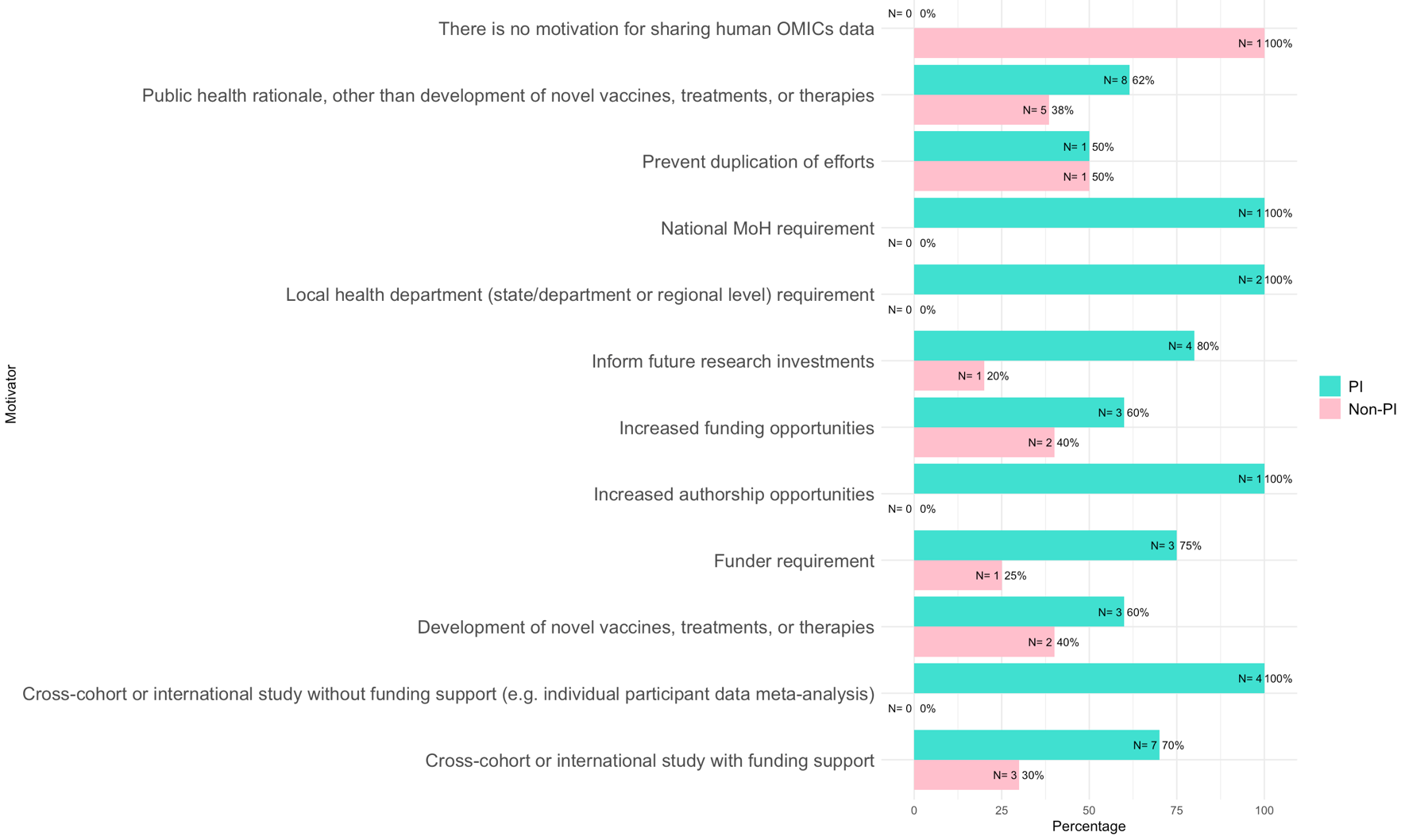


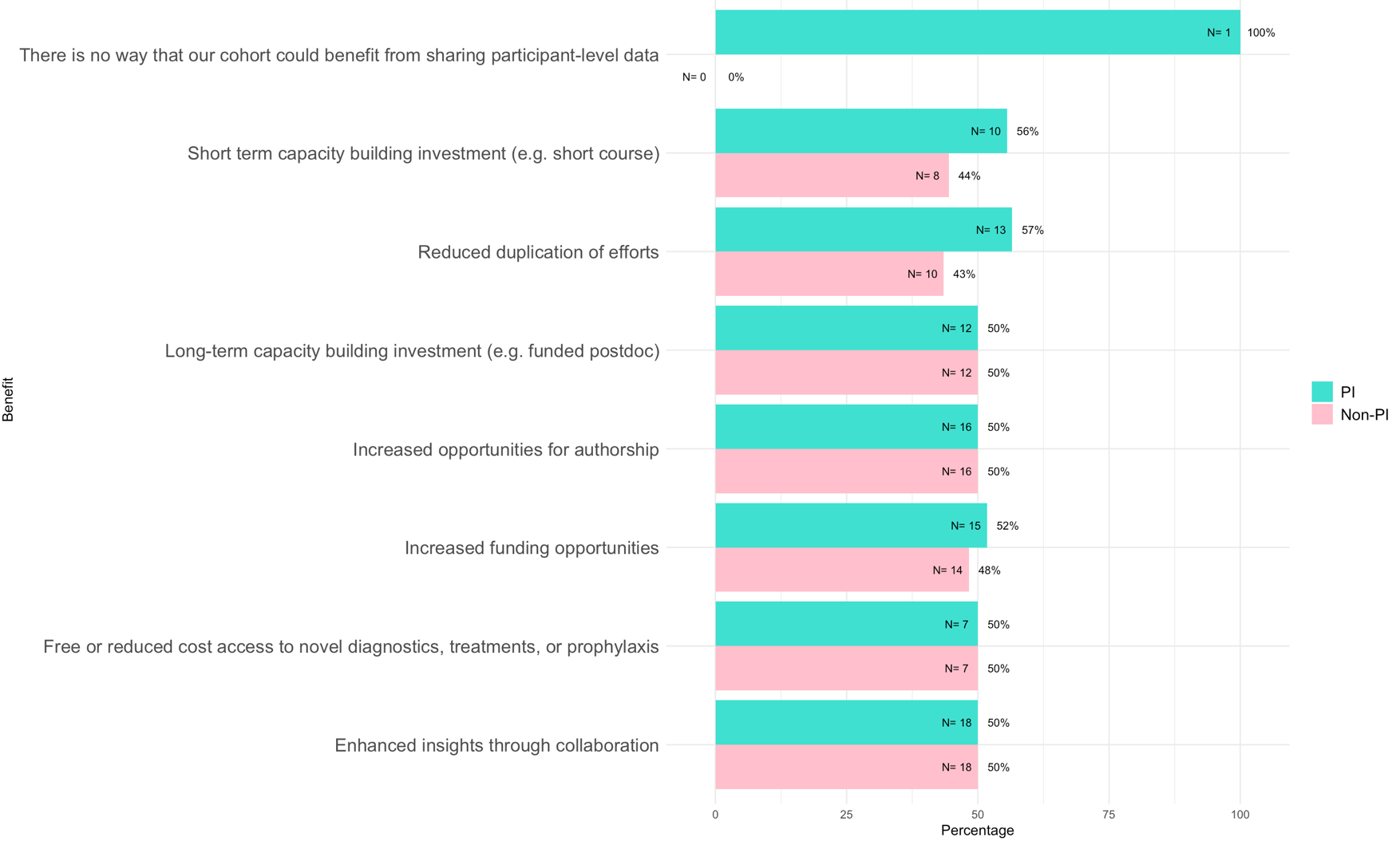
**Appendix Figure 2A. Comparison of benefits of sharing participant-level clinical-epidemiological data by respondent role in the cohort (PI vs non-PI)**

**
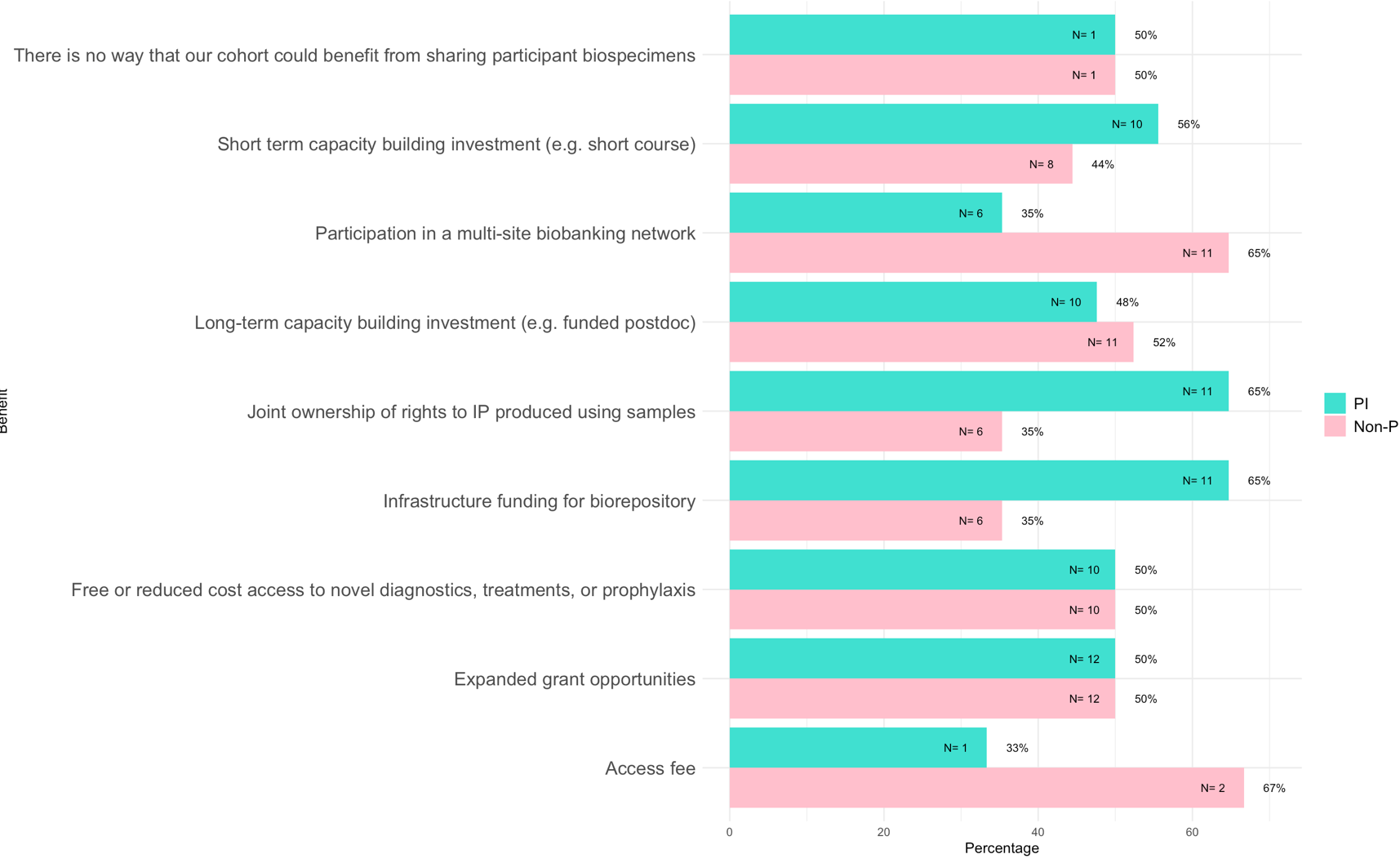
Appendix Figure 2B. Comparison of benefits of sharing human biological samples by respondent role in the cohort (PI vs non-PI)**

Benefits

Benefits

**
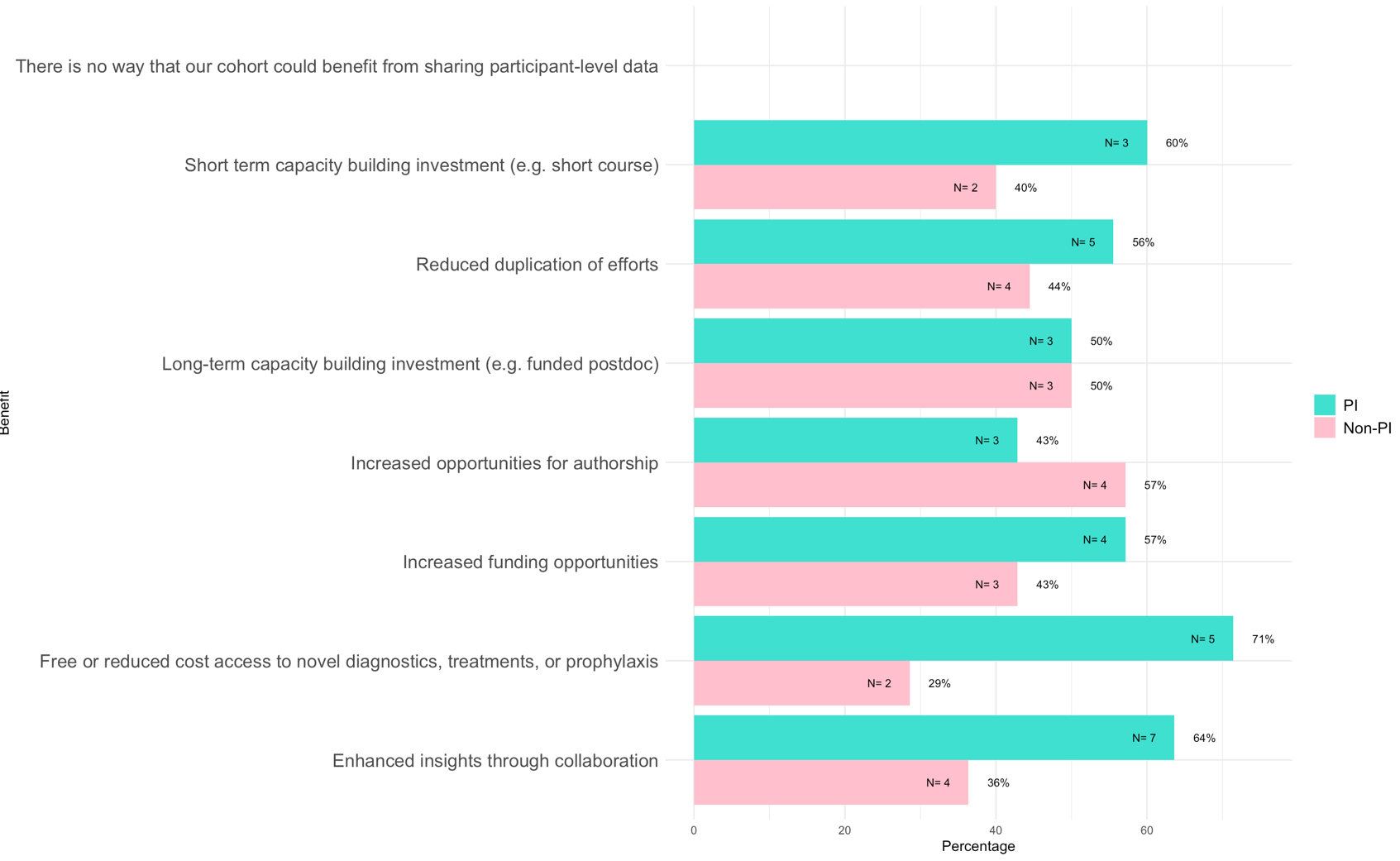
Appendix Figure 2C. Comparison of benefits of sharing human omics data by respondent role in the cohort (PI vs non-PI)**

Benefits

**
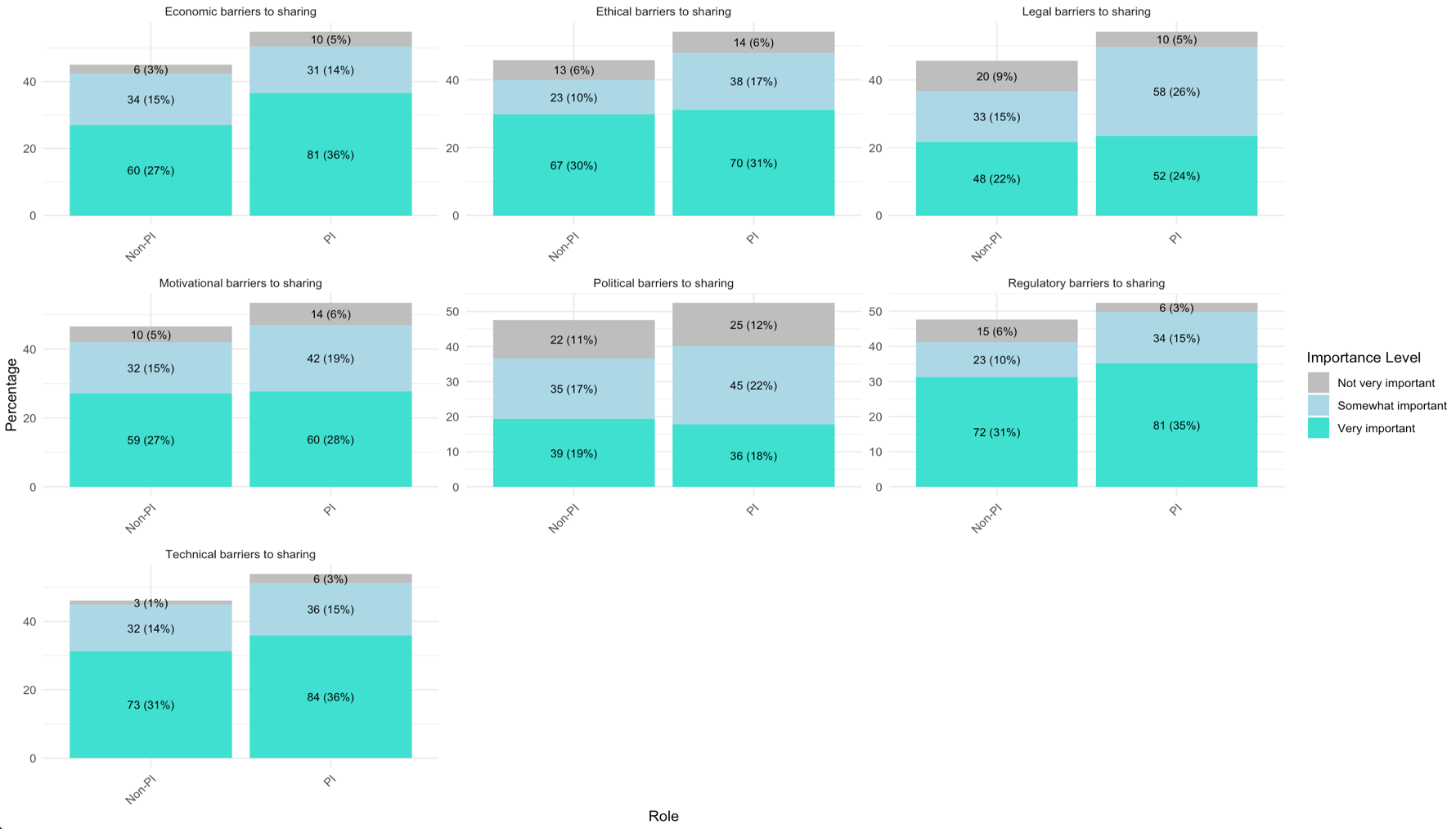
Appendix Figure 3. Barriers to sharing by respondent role in the cohort (PI vs non-PI)**

**Appendix Figure 4. Barriers to sharing data and samples by respondent role in the cohort (PI vs non-PI) and data type**

**Appendix Figure 5A. Barriers to sharing data and samples during epidemics by respondent role in the cohort (PI vs non-PI)**

**Appendix Table 1. Number of respondents to barriers questions in Appendix Figure 5A.**

| N | Economic barriers | Ethical barriers | Legal barriers | Motivational barriers | Political barriers | Regulatory barriers | Technical barriers |
| --- | --- | --- | --- | --- | --- | --- | --- |
| Clin-epi data | | | | | | | |
| PI | 31 | 32 | 32 | 28 | 26 | 30 | 33 |
| Non-PI | 25 | 26 | 25 | 25 | 22 | 27 | 27 |
| Human genetic data | | | | | | | |
| PI | 7 | 7 | 7 | 7 | 7 | 7 | 7 |
| Non-PI | 3 | 3 | 3 | 3 | 3 | 3 | 4 |
| Human biological samples | | | | | | | |
| PI | 28 | 28 | 24 | 27 | 23 | 27 | 29 |
| Non-PI | 20 | 21 | 20 | 20 | 21 | 23 | 22 |

**Appendix Figure 5B. Barriers to sharing data and samples outside of epidemic settings according to respondent role in the cohort (PI vs non-PI)**

**Appendix Table 2. Number of respondents to barriers questions in Appendix Figure 5A.**

| N | Economic barriers | Ethical barriers | Legal barriers | Motivational barriers | Political barriers | Regulatory barriers | Technical barriers |
| --- | --- | --- | --- | --- | --- | --- | --- |
| Clin-epi data | | | | | | | |
| PI | 26 | 25 | 25 | 24 | 22 | 26 | 26 |
| Non-PI | 23 | 23 | 24 | 26 | 22 | 25 | 27 |
| Human genetic data | | | | | | | |
| PI | 9 | 10 | 10 | 9 | 9 | 10 | 9 |
| Non-PI | 3 | 3 | 3 | 3 | 3 | 3 | 3 |
| Human biological samples | | | | | | | |
| PI | 26 | 27 | 27 | 23 | 25 | 28 | 25 |
| Non-PI | 21 | 20 | 21 | 22 | 19 | 22 | 22 |
